# Thermal Performance Curves of Multiple Isolates of *Batrachochytrium dendrobatidis*, a Lethal Pathogen of Amphibians

**DOI:** 10.3389/fvets.2021.687084

**Published:** 2021-06-22

**Authors:** Ciara N. Sheets, Deena R. Schmidt, Paul J. Hurtado, Allison Q. Byrne, Erica Bree Rosenblum, Corinne L. Richards-Zawacki, Jamie Voyles

**Affiliations:** ^1^Department of Biology, University of Nevada, Reno, NV, United States; ^2^Department of Mathematics and Statistics, University of Nevada, Reno, NV, United States; ^3^Department of Environmental Science, Policy, and Management, University of California, Berkeley, Berkeley, CA, United States; ^4^Museum of Vertebrate Zoology, University of California, Berkeley, Berkeley, CA, United States; ^5^Department of Biological Sciences, University of Pittsburgh, Pittsburgh, PA, United States

**Keywords:** amphibian declines, chytridiomycosis, *Batrachochytrium dendrobatidis*, thermal performance curves, climate, latitudinal gradient

## Abstract

Emerging infectious disease is a key factor in the loss of amphibian diversity. In particular, the disease chytridiomycosis has caused severe declines around the world. The lethal fungal pathogen that causes chytridiomycosis, *Batrachochytrium dendrobatidis* (*Bd*), has affected amphibians in many different environments. One primary question for researchers grappling with disease-induced losses of amphibian biodiversity is what abiotic factors drive *Bd* pathogenicity in different environments. To study environmental influences on *Bd* pathogenicity, we quantified responses of *Bd* phenotypic traits (e.g., viability, zoospore densities, growth rates, and carrying capacities) over a range of environmental temperatures to generate thermal performance curves. We selected multiple *Bd* isolates that belong to a single genetic lineage but that were collected across a latitudinal gradient. For the population viability, we found that the isolates had similar thermal optima at 21°C, but there was considerable variation among the isolates in maximum viability at that temperature. Additionally, we found the densities of infectious zoospores varied among isolates across all temperatures. Our results suggest that temperatures across geographic point of origin (latitude) may explain some of the variation in *Bd* viability through vertical shifts in maximal performance. However, the same pattern was not evident for other reproductive parameters (zoospore densities, growth rates, fecundity), underscoring the importance of measuring multiple traits to understand variation in pathogen responses to environmental conditions. We suggest that variation among *Bd* genetic variants due to environmental factors may be an important determinant of disease dynamics for amphibians across a range of diverse environments.

## Introduction

Emerging infectious diseases are a primary driver of global amphibian declines (1). Disease outbreaks from ranaviruses, chytrid fungi, and bacterial pathogens have contributed to an unprecedented loss of global amphibian diversity (2–4). Therefore, understanding what factors influence the emergence, spread, pathogenicity, and ecology of these pathogens is important for amphibian conservation (5). Many of these pathogens are strongly influenced by their local environments, and corresponding shifts in pathogen phenotypic traits (e.g., reproductive rates, pathogen persistence in the environment) can alter disease risks for susceptible amphibian host species (6–8). By investigating how a pathogen responds to its environment, as well as the genotypic and phenotypic variation that underpins those responses, we can begin to unravel the disease dynamics that threaten amphibians (9, 10).

Chytridiomycosis is one such infectious disease that is lethal to many amphibian species and has caused global declines in susceptible species (1, 11). The disease is caused by the fungal pathogens, *Batrachochytrium dendrobatidis* (*Bd*) (12) and *Batrachochytrium salamandrivorans* (*Bsal*) (13). However, *Bd* has spread globally and impacted far more amphibian host species than *Bsal*, making it a priority pathogen for study (1). Since its discovery in 1999, *Bd* has spread rapidly through multiple naïve amphibian communities, causing mass mortality events, and even the complete extinction of amphibian species (11). No other pathogen is known to have had such a ubiquitous effect on such a broad range of host species and in so many different environments (1, 14, 15). As a result, *Bd*-related declines have been called, “the most spectacular loss of biodiversity due to disease in recorded history” (11).

*Bd* has a two-stage life cycle that consists of a substrate-dependent immobile sporangium and a free-living uniflagellated, motile zoospore (12, 16). Infection occurs during the motile zoospore stage of the pathogen's life cycle (12, 16). The motile zoospores encyst on a substrate, such as the keratinized tissue found in amphibian larval mouthparts or on adult epidermis, and then mature into a zoosporangium (12, 17, 18). Zoosporangia produce motile zoospores and then release the new zoospores into the environment to re-infect the same host or transmit to another individual host (18). Once infection is established within a host, increases in infection intensity (or pathogen load) in amphibian skin is a key feature of pathogenesis (19, 20). As such, understanding the factors that regulate *Bd* growth and reproductive rates is integral to resolving questions concerning pathogenesis and the disease ecology of this lethal disease system (21, 22).

Recent phylogenetic analyses indicate that there are several major lineages of *Bd* that are genetically distinct (23–25). One lineage that has garnered considerable attention from the scientific community, due to its high lethality, is the Global Panzootic Lineage (*Bd*GPL) (23, 24, 26). Genomic sequencing of many *Bd* isolates within this lineage has shown that it contains substantial genetic diversity, including two genetic clades (*Bd*GPL1 and *Bd*GPL2) (26–28). With a global distribution, *Bd*GPL occurs in a wide range of amphibian habitats and causes disease in diverse microclimates and thermal environments (2, 29). As such, researchers have focused on resolving the factors that determine variation among *Bd*GPL isolates to understand how temperature may mediate disease dynamics (21, 22, 30, 31). To date, no clear patterns have emerged that can explain the extent of variation among and within *Bd*GPL isolates across diverse thermal environments. This outstanding question may be most appropriately investigated by generating thermal performance curves, which would allow for comparative investigations within the *Bd*GPL lineage.

**Figure 1:**
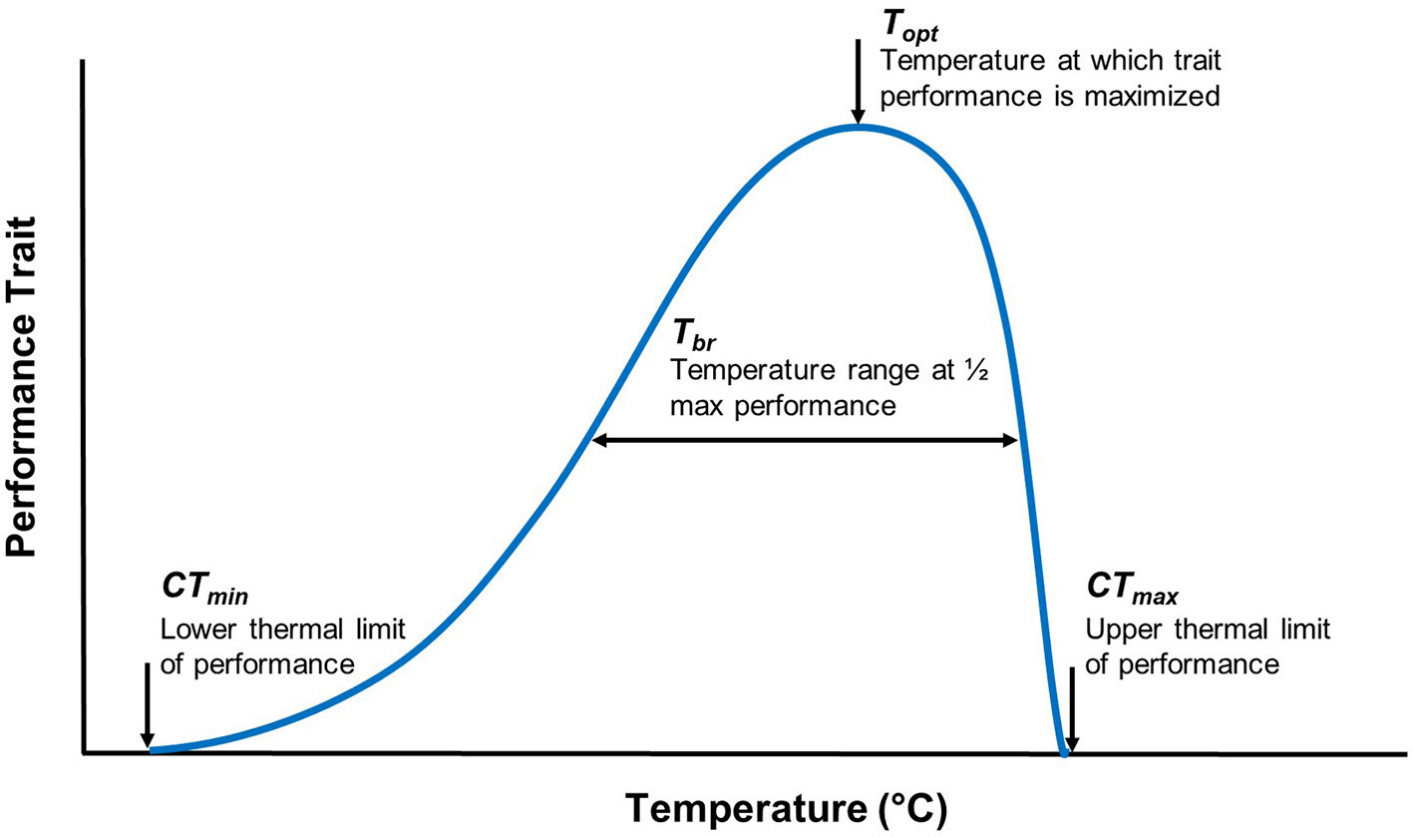
Traditional thermal performance curve parameters for a given trait. When a performance curve is generated, the performance of a trait is plotted against a temperature range. The thermal breadth (*T*_*br*_), also referred to as the thermal tolerance range, is the temperature range at which a level of performance is achieved. A thermal optimum (*T*_*opt*_) is the temperature at which trait performance is maximized. The critical thermal minimum (*CT*_*min*_) and maximum (*CT*_*max*_) are the lower and upper thermal limits of a trait's performance, respectively.

**Figure 2:**
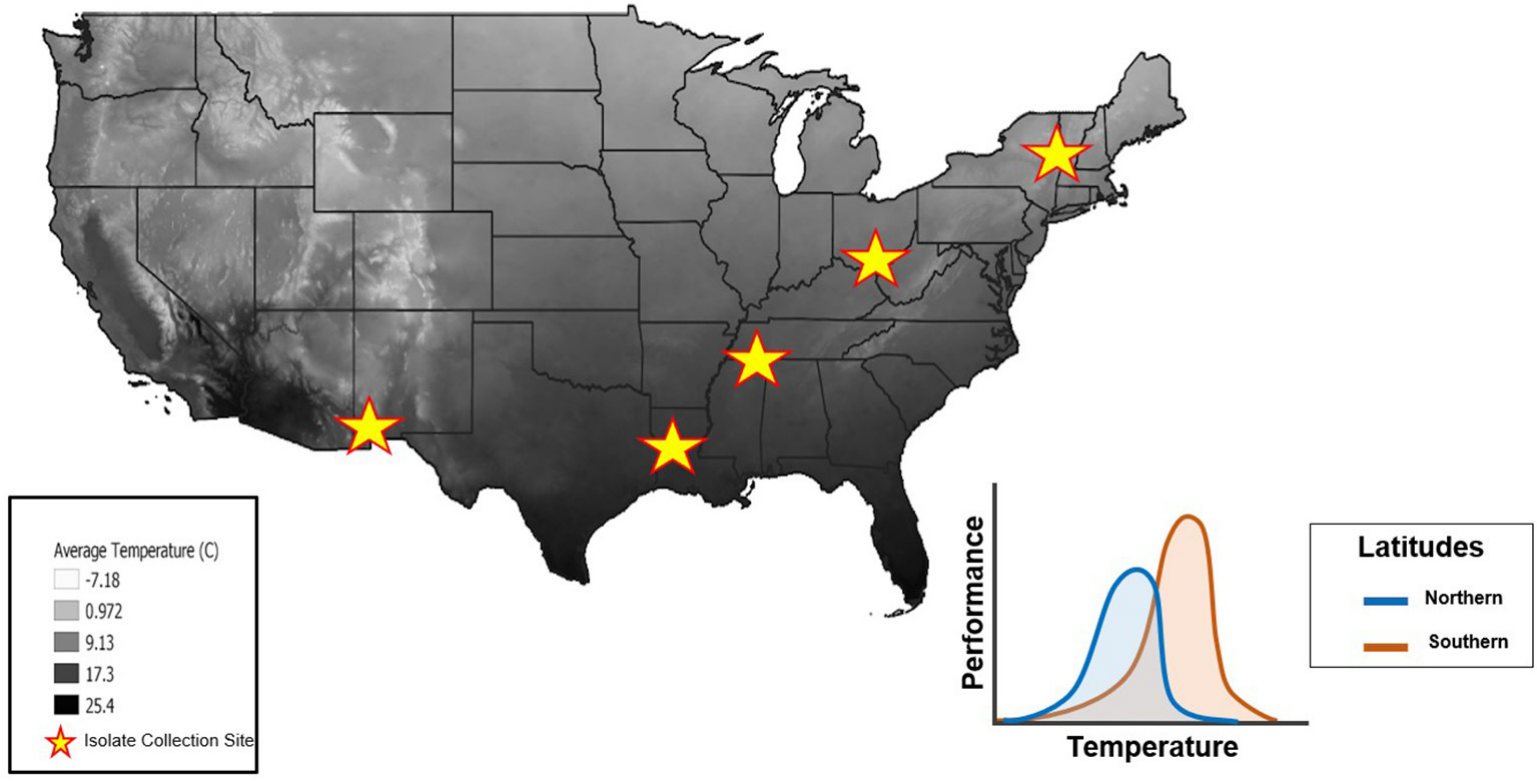
Mean annual air temperatures throughout the United States (highest temperatures in black, lowest temperatures in white). Stars represent the locations where isolates were collected from across a latitudinal gradient. The thermal constraint hypothesis (“hotter is better”) predicts that isolates from Northern latitudes will have lower maximal performance at a lower *T*_*opt*_(blue curve) whereas isolates from Southern latitudes will have a higher maximal performance at a higher *T*_*opt*_ due to adaptations from local temperature regimes (orange curve). Temperature data from the National Forest Climate Change Maps website to generate this figure in QGIS software (41).

Thermal performance curves (TPCs) are widely used to measure an organism's performance across a range of temperatures, estimate the thermal sensitivity of different traits, and facilitate an understanding of ecological and evolutionary processes that may explain an organism's success within a given environment (32–34). TPCs include measures of thermal optimum [i.e., temperature optimum (*T*_*opt*_)], critical thermal minimum (*CT*_*min*_), critical thermal maximum (*CT*_*max*_), and thermal tolerance range (also known as thermal breadth; *T*_*br*_) (Figure 1). Temperature sensitive parameters that determine an organism's TPC frequently vary with geographic clines (e.g., latitude), reflecting local adaptation (34, 35). TPC models (e.g., vertical or horizontal shifts) offer a framework to consider the adaptive potential for temperature-sensitive organisms (36, 37). For example, horizontal shifts toward a higher *T*_*opt*_ would provide evidence in support of the “hotter is better” hypothesis, which predicts that organisms will adapt to thermal conditions according to thermodynamic constraints (e.g., with higher *T*_*opt*_ in latitudes where mean temperatures are higher) (38–40) (Figure 2).

It is generally thought that *Bd* has a thermal tolerance range of 2–28 °C (42), with a *T*_*opt*_ of 17–25 °C (8, 43), and *CT*_*min*_, and *CT*_*max*_ of 2–5 and 25–28 °C, respectively (42, 44). Mounting evidence suggests that *Bd* isolates differ in their thermal optima (8, 42, 44), but experimental approaches have not yet explored this idea by comparing isolates collected across a latitudinal gradient (45). We predicted that the TPCs of *Bd* isolates collected along a latitudinal gradient would differ due to thermal constraints in each region. More specifically, we expected that isolates from northern latitudes would have a lower *T*_*opt*_ and exhibit a lower maximum performance at that temperature. In contrast, we expected isolates from southern latitudes to have a higher *T*_*opt*_ and higher performance at that temperature (Figure 2). To test these predictions, we generated TPCs for five *Bd* isolates collected across a latitudinal gradient.

## Materials and Methods

### *Bd* Isolate Collection and Maintenance

We used five different *Bd* isolates that originated from amphibians in the United States (Table 1). The collection of *Bd* isolates from the United States provides an ideal repertoire for investigating phenotypic variation and differences in TPCs for multiple reasons. First, the collection of *Bd* isolates that originate from amphibians in the United States is large, with numerous isolates from across the country, spanning a latitudinal gradient. Second, previous work using a microfluidic PCR genotyping method (one that targets ~200 loci) suggested that *Bd*GPL is the primary lineage found in North America (25).

**Table 1:** Genotyping for isolates of *Batrachochytrium dendrobatidis*, amphibian host, and geographic origins.

**Genotype**	**Isolate**	**Location**	**Host species name**	**Latitude**
GPL1	Louisiana (LA)	New Orleans, LA	*Acris crepitans*	29.9511 °N
GPL1	Tennessee (TN)	Memphis, TN	*Lithobates sphenocephala*	35.1495 °N
GPL1	Vermont (VT)	VT	*Lithobates clamitans*	44.5588 °N
GPL2	New Mexico (NM)	Beaver Creek, NM	*Lithobates catesbeianus*	34.5199 °N
GPL2	Ohio (OH)	Toledo, OH	*Lithobates pipiens*	41.6528 °N

All isolates were cryoarchived and subsequently revived according to standard protocols (46) prior to the beginning of the experiment. Following isolate revival, we cultured the *Bd* isolates in tryptone/gelatin hydrolysate/lactose (TGhL) liquid growth media in 75 cm^2^ tissue culture flasks (47). We incubated each isolate at 21°C and monitored them through the *Bd* life cycle until the point of peak zoospore densities (42). Once each culture flask reached peak zoospore density, 2 mL of culture was transferred to a new culture flask containing 13 mL of fresh TGhL media for standard passage. We used a biosafety cabinet for all laboratory work involving these isolates (e.g., passaging, experimental setup).

### Generating Thermal Performance Curves

We filtered each of the five cultures using sterile filter paper (Whatman Qualitative Filter Papers, Grade 3) and used a vacuum filtration pump to remove zoosporangia (47). With the remaining filtrate, we quantified zoospores using a hemocytometer and diluted each culture with TGhL to a concentration of ~50 × 10^4^ zoospores/mL (48). We inoculated the cultures of each isolate containing only zoospores into 96-well-plates. We then added 50 μL of additional TGhL media to each well. We included five negative control wells with 50 μL of 50 × 10^4^ zoospores/mL heat-killed zoospores and 50 μL of TGhL media for each isolate (48). We filled the perimeter wells of the plate with 150 μL TGhL media to provide a buffer against culture evaporation (45).

To establish a thermal profile for each respective isolate, we incubated all isolates at multiple stable temperatures (4, 12, 17, 21, 25, 26, and 27 °C). Because we used only zoospores to start the growth experiments, we were able to track and quantify several parts of the *Bd* life cycle as they occurred at different time points in these different temperature conditions. Specifically, by tracking cultures for multiple successive days, we were able to measure the change in population growth, time to maximum zoospore densities, zoospore densities, and calculate fecundity (49). At multiple time points following experimental set up (Day 0), we randomly selected five wells (*N* = 5) for each of two destructive measures: zoospore counts and viability assays (49).

To quantify zoospore densities, we manually withdrew 20 μL of culture and counted live zoospores using a hemocytometer (48). Following these counts, we omitted those wells for the remainder of the experiment (48). To measure population growth, we conducted a standard viability assay (45). The MTT viability assay is a standard microbiological technique where a yellow tetrazolium salt 3-(4,5-dimethylthiazol-2-yl)-2,5-diphenyltetrazolium bromide (MTT) is reduced to purple MTT-formazan crystals in metabolically active cells (50). These crystals can be solubilized, and the color change can be quantified by reading culture absorbance at 570 nm (45). We added 20 μL of MTT to each experimental and negative control wells of the plate selected for that day and incubated the plate at 21°C for 2 h (45). After incubation, we added 140 μL of the stop-reagent to stop the reaction and solubilize the MTT-formazan crystals (45). We then read culture absorbance at 570 nm using a Biotek EL x 800 Absorbance Reader.

### *Bd* Isolate Genotyping

We genotyped the isolates using an amplicon sequencing approach according to published protocols (51). Briefly, we extracted DNA following the manufacturer's protocol for the Qiagen DNeasy Blood and Tissue kit. Next, to prepare raw DNA extracts for sequencing, we cleaned each using an isopropanol precipitation and preamplified each in two separate PCR reactions, each containing 96 primer pairs. Primers were designed to target 150–200 base pair regions of the *Bd* nuclear and mitochondrial genome (51). After preamplification, samples were cleaned using EXOSap-it™ (ThermoFisher Scientific) and diluted 1:5 in water. Finally, we cleaned and diluted products from the two preamplification reactions, combined in equal proportions, and sent to the University of Idaho IBEST Genomics Resources Core, where they were loaded into a Fluidigm June LP 192.24 IFC (Fluidigm Inc.) for amplification and barcoding. Amplified products were pooled and sequenced on an Illumina MiSeq.

Raw sequences were processed as previously described (25, 51). Raw reads were joined via FLASH [(52); v.1.2.11] and consensus sequences for each sample/amplicon combination were called using the reduce amplicons R script (https://github.com/msettles/dbcAmplicons/blob/master/scripts/R/reduce_amplicons.R). Here, consensus sequences use IUPAC ambiguity codes to indicate multiple alleles at a locus. We compared the consensus sequences of each of our five isolates to 21 previously published *Bd* samples using a phylogenetic approach. We selected previously published reference sequences to represent every known major *Bd* lineage (25). To create a phylogeny, we used a gene tree to species tree approach: first aligning all sequences for each amplicon using MUSCLE [(53); v.3.32], then creating a tree for each amplicon using RAxML [(54); v.8.2.11] to search for the best scoring ML tree from 100 bootstrap replicates. Afterwards, we used newick utils [(55); v.1.6] to collapse all nodes in each amplicon tree with <10 bootstrap support. We then input a total of 190 amplicon trees with collapsed branches into Astral-III [(56); v.5.5.9], which estimates an unrooted species tree given a set of unrooted gene trees using the multispecies coalescent model.

### Statistical Analysis

For all statistical analyses, we used R version 3.4.3 (57). We used QGIS software and the “ggplot2” package within R to generate figures. Summary statistics reported in the figures and the tables include means ± standard error (SE) of the viability, zoospore densities, or fecundity measure among isolates or between genotypes. We analyzed the performance of each isolate when grouped by genetic variant and independently to compare for differences among isolates at *T*_*opt*_, *CT*_*min*_, and *CT*_*max*_ temperature treatments. We used Analysis of Variance (ANOVA) and Games Howell *post-hoc* tests to make comparisons in mean maximum viability (OD following the MTT assay), mean maximum zoospore densities, mean fecundity, and time to maximum zoospore densities. We used a non-parametric *post-hoc* test when there was a violation of the homogenous variance assumption for each of the traits compared among isolates. To calculate mean maximum viability and maximum zoospore densities, we used the measures from within the 2–6 day period at which cultures exhibited maximum viability or zoospore densities in each temperature condition.

To make comparisons of fecundity, we calculated the ratio of zoospores densities to mean culture viability. Within the fecundity calculations, all viability measurements that were <0.005 were considered zeros to ensure that fecundity ratios were not artificially inflated. For statistical analyses, we log-transformed the fecundity metric and added a correction factor of 1 to accommodate for the wells that had zero zoospores. For comparing genetic variants in viability, zoospore densities, fecundity, and time to maximum zoospore densities, we used Welch's *t*-test because we had unequal variance after grouping by genotype. We used a Bonferroni correction after running the *t*-test at each temperature experiment for comparisons of MTT across the thermal range to reduce the likelihood of a type-1 error.

To further quantify the differences across temperatures, we fit a logistic growth curve to the normalized optical density measurements (i.e., *Bd* viability) data time series for each isolate-temperature combination. This approach allowed us to estimate the intrinsic growth rate (*r*) and carrying capacity (*K*). We used the resulting estimates for *r* and *K* to quantify differences among isolates over the range of temperatures considered. To calculate 95% confidence intervals for these estimates, we used likelihood profile-based methods (58, 59). We attempted to constrain *r* estimates to follow a Johnson–Lewin (J–L) curve as a function of temperature to characterize the thermal breadth of each isolate (59). See the Supplementary Material for details.

## Results

### Differential Responses to Temperature Between Genetic Variants

**Figure 3:**
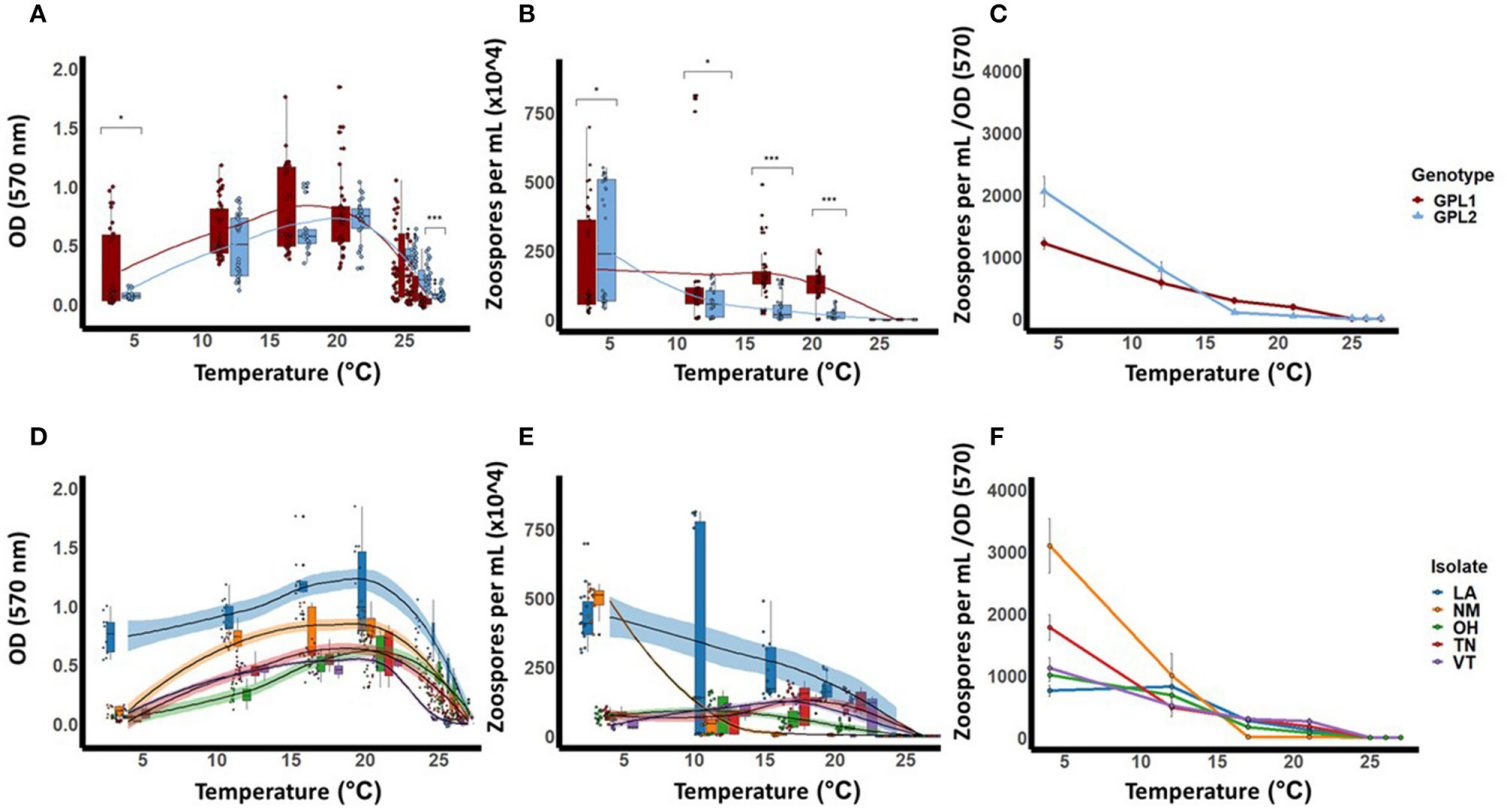
Thermal performance curves for isolates of *Batrachochytrium dendrobatidis* (*Bd*). Five isolates were collected from across a latitudinal gradient in the United States, genotyped, and tested for these responses across the known thermal range for *Bd* (4–27 °C). Data show means (±SE) for *Bd* viability **(A)**, zoospore densities **(B)**, and fecundity (calculated as optical density/zoospore densities) **(C)**, for two *Bd* genotypes, Global Panzootic Lineage 1 and Global Panzootic Lineage 2 (*Bd*GPL1 and *Bd*GPL2; top row, red and blue). Additionally, data show means (±SE) for *Bd* viability **(D)**, zoospore densities **(E)**, and fecundity **(F)**, for each of the five isolates that were collected in Louisiana (LA, blue), New Mexico (NM, organe), Ohio (OH, green), Tennessee (TN, red), and Vermont (VT, purple). Significance levels are indicated by the asterisks as such: * < 0.05, ** < 0.01, *** < 0.001.

Our genetic sequencing revealed that these isolates belong to the *Bd*GPL clades 1 or 2 (Table 1). When we grouped the isolates by genetic lineage, we found no differences in viability between *Bd*GPL1 and *Bd*GPL2 lineages at the *T*_*opt*_, 21°C [*t*_(57.51)_ = 0.91, *p* = 0.37; Figure 3A]. Furthermore, there were no significant differences in viability between *Bd*GPL1 and *Bd*GPL2, except at the low temperature of 4 °C and the high temperature at 27 °C (Figure 3A, Table 2).

**Table 2:** Population growth viability (MTT) across temperatures (means, SE, *r*, and *K*).

	**Isolate ID**	**4** ^****°****^**C**			**12** ^****°****^**C**			**17** ^****°****^**C**			**21** ^****°****^**C**			**25** ^****°****^**C**			**26** ^****°****^**C**			**27** ^****°****^**C**		
		**Mean (±SE)**	***r***	***K***	**Mean (±SE)**	***r***	***K***	**Mean (±SE)**	***r***	***K***	**Mean (±SE)**	***r***	***K***	**Mean (±SE)**	***r***	***K***	**Mean (±SE)**	***r***	***K***	**Mean (±SE)**	***r***	***K***
GPL1	LA	0.75 ± 0.053	0.2591	0.6246	0.91 ± 0.036	0.5867	0.8337	1.19 ± 0.050	0.8546	1.275	1.12 ± 0.104	1.504	1.048	0.78 ± 0.046	0.9155	0.9878	0.40 ± 0.049	0.482	107300	0.02 ± 0.007	0.4817	0.0462
	VT	0.09 ± 0.013	0.1644	2.022	0.46 ± 0.016	0.2312	0.4757	0.47 ± 0.021	0.4321	0.6248	0.53 ± 0.011	0.3991	0.602	0.04 ± 0.003	0.9506	0.0716	0.02 ± 0.004	5.431	0.0035	0.00 ± 0.001	1.433	5.725
	TN	0.02 ± 0.002	0.07248	3.661	0.45 ± 0.019	0.8495	0.4357	0.57 ± 0.027	0.7361	0.6317	0.63 ± 0.052	0.8277	0.6702	0.23 ± 0.019	1.017	0.3259	0.14 ± 0.018	1.314	0.1533	0.06 ± 0.005	1.874	0.0533
GPL2	NM	0.10 ± 0.015	0.1939	0.1047	0.74 ± 0.023	0.6297	0.5608	0.76 ± 0.076	0.8781	0.8241	0.82 ± 0.029	0.808	0.882	0.40 ± 0.018	1.324	0.4455	0.21 ± 0.021	1.187	0.2709	0.07 ± 0.007	1.063	0.075
	OH	0.06 ± 0.005	0.1305	0.0996	0.25 ± 0.019	0.3071	0.2313	0.52 ± 0.029	0.9106	0.5445	0.60 ± 0.046	0.8264	0.6487	0.44 ± 0.036	1.498	0.3906	0.21 ± 0.032	1.451	0.2994	0.10 ± 0.012	0.9965	0.1123

We also measured zoospore densities for the two genetic lineages in all temperatures because the capacity to generate high zoospore densities is thought to be a critical factor for disease development (21). We found patterns in our measures of zoospore densities that differed from those in our viability assays (Figure 3B). There were significant differences between *Bd*GPL1 and *Bd*GPL2 in zoospore densities at every temperature where zoospores were produced (Figure 3B, Table 3). Specifically, *Bd*GPL1 had higher zoospore densities than *Bd*GPL2 at all temperatures except 4 °C (Table 3). We found that fecundity was significantly different between *Bd*GPL1 and *Bd*GPL2 at three temperatures: 4 °C [*t*_(182.81)_ = −3.2, *p* = 0.002], 17 °C [*t*_(162.63)_ = 6.039, *p* ≤ 0.001], and 21 °C [*t*_(131.85)_ = 6.9127, *p* ≤ 0.001] (Figure 3C). There were no significant differences between *Bd*GPL1 and *Bd*GPL2 in the time to maximum zoospore densities at any temperature except 21 °C [*t*_(22.29)_ = −2.7584, *p* = 0.01].

**Table 3:** Zoospore density descriptive difference of means using *t*-tests between genotypes.

	**4** ^****°****^**C**			**12** ^****°****^**C**			**17** ^****°****^**C**			**21** ^****°****^**C**			**25** ^****°****^**C**			**26** ^****°****^**C**			**27** ^****°****^**C**		
	***t***	**df**	***p***	***t***	**df**	***p***	***t***	**df**	***p***	***t***	**df**	***p***	***t***	**df**	***p***	***t***	**df**	***p***	***t***	**df**	***p***
Genotype	−2.0148	56	0.049	2.4708	51	0.017	8.035	67.4	<0.001	11.56	54.3	<0.001	NA	NA	NA	NA	NA	NA	NA	NA	NA

### Differential Responses to Temperature Among the *Bd* Isolates

All isolates exhibited maximum viability at 21°C. However, there were differences among isolates in their mean viability at the *T*_*opt*_ of 21°C [ANOVA, *F*_(4,63)_ = 15.94, *P* < 0.001, Table 2, Figure 3D]. The isolate from Louisiana exhibited the greatest mean viability in the *T*_*opt*_, 21°C (Table 2, Figure 3D), as well as at every other temperature treatment except 27 °C (Table 2, Figure 3D). The isolate from Vermont exhibited the lowest viability except in the low temperature treatments of 4 and 12 °C (Table 2, Figure 3D).

We found that there were differences among the *Bd* isolates in their viability in both low and high temperature conditions (Table 2, Figure 3D). For the lowest temperature treatment, all isolates exhibited minimal growth at 4 °C but there were differences among the isolates in viability at that temperature [Table 2; ANOVA, *F*_(4,45)_ = 146.6, *P* < 0.001]. The isolates also differed in their responses to high temperature treatments (Table 2, Figure 3D). The isolate from Ohio exhibited significantly greater viability at the highest temperature treatment of 27 °C [ANOVA, *F*_(4,70)_ = 25.82, *P* < 0.001; Game's Howell, *P* < 0.01], whereas the isolate from Vermont had low viability at 26 °C and was not viable at 27 °C (Figure 3D).

The patterns found in zoospore densities among isolates also differed from viability results (Figure 3E). Specifically, two of the isolates produced their maximum zoospore densities at the low temperatures of 4 and 12 °C (Figure 3E, Table 4). Notably, for the New Mexico isolate, zoospore densities were highest at 4 °C and were dramatically lower at all other temperatures (Table 4, Figure 3E). Accordingly, the New Mexico isolate exhibited the highest fecundity (zoospores per viability measure) at 4 °C (Figure 3F). All isolates exhibited a similar pattern, with higher fecundity in lower temperatures, but it was most pronounced in the New Mexico isolate at 4 °C. In addition, we found that the time to maximum zoospore densities differed among isolates at 4 °C [ANOVA, *F*_(4,70)_ = 250.9, *P* < 0.001] and 21°C [ANOVA, *F*_(4,70)_ = 48.36, *P* < 0.001]. We also found that, although the cultures were viable and growth measurements increased at the higher temperatures of 25, 26, and 27 °C, none of the isolates produced zoospores at these high temperatures (Figure 3E).

**Table 4:** Zoospore densities across temperatures (means, SE, *r*, and *K*).

	**Isolate ID**	**4 ^**°**^C**	**12 ^**°**^C**	**17 ^**°**^C**	**21 ^**°**^C**	**25 ^**°**^C**	**26 ^**°**^C**	**27 ^**°**^C**
		**Mean (±SE)**	**Mean (±SE)**	**Mean (±SE)**	**Mean (±SE)**	**Mean (±SE)**	**Mean (±SE)**	**Mean (±SE)**
GPL1	LA	431.9 ± 27.141	314.7 ± 92.641	235.7 ± 27.795	172.8 ± 10.806	NA	0	0
	VT	38.9 ± 3.998	89.1 ± 5.996	127.3 ± 4.525	84.6 ± 16.238	NA	0	0
	TN	78.4 ± 4.296	56.1 ± 10.333	119.7 ± 17.980	120.6 ± 9.395	NA	0	0
GPL2	NM	492.1 ± 13.133	37.4 ± 6.995	4.5 ± 0.885	2.8 ± 0.433	NA	0	0
	OH	70.3 ± 4.853	89.2 ± 16.559	65.7 ± 11.233	30.7 ± 3.596	NA	0	0

**Figure 4:**
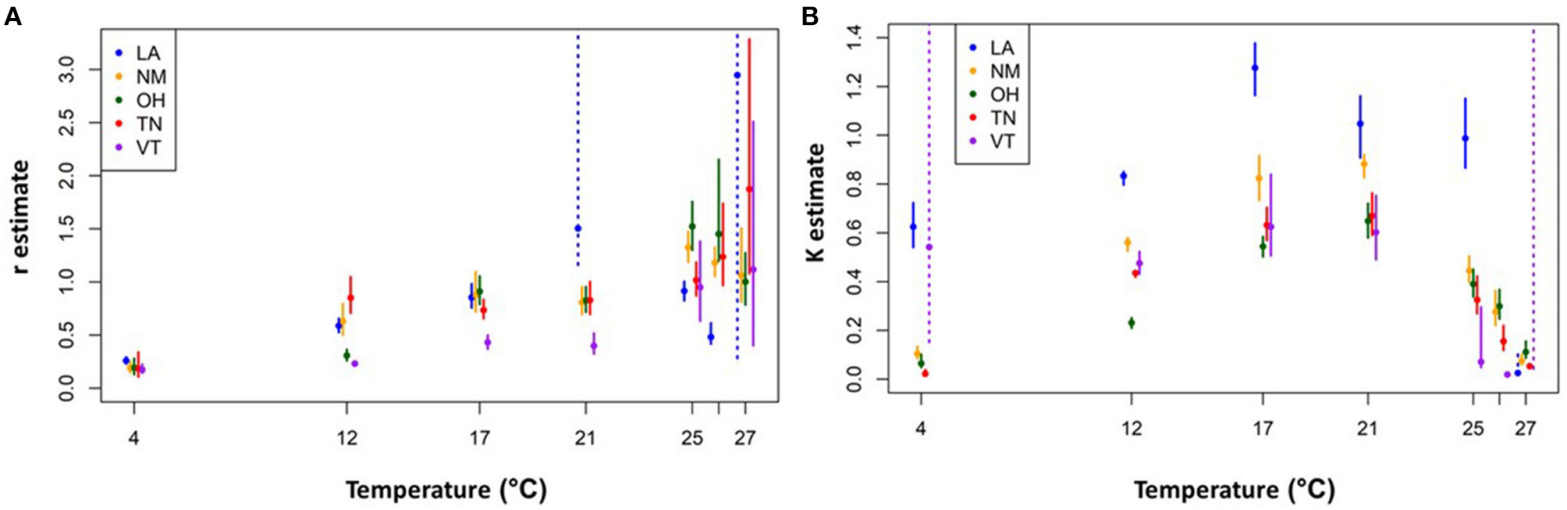
Logistic growth curve parameter estimates for each isolate and temperature combination. Intrinsic exponential growth rate (*r*) estimates **(A)** carrying capacity (*K*) estimates **(B)**. Solid circles indicate the best fit parameter values and vertical bars show the 95% confidence intervals. The dashed vertical bars indicate situations in which the confidence interval for the parameter of interest had a poorly resolved upper end point due to parameter identifiability issues (e.g., for time series data in the exponential growth phase, all values of *K* above a certain threshold will give equally good fits). Not shown, for clarity: VT-26 °C *r* estimate is 8.58, CI = (4.61, 14.12). See Supplementary Material for further details.

We then assessed how these *r* and *K* estimates varied with temperature for each isolate. The overall trend for all isolates is *r* estimates that increase and then plateau for temperatures up to 21 °C (Figure 4A). However, for higher temperatures (25–27 °C), the *r* estimates are larger and more variable both across isolates and in terms of having larger confidence intervals. The intrinsic growth rates at the higher temperatures, however, do not yield much long-term growth. The corresponding *K* estimates also increase and plateau at ~21°C, but then markedly decline at the higher temperatures (Figure 4B). The combined effect is a short-lived exponential growth phase that quickly reaches a relatively low upper bound at these high temperatures (Supplementary Materials).

## Discussion

Chytridiomycosis is a disease that has impacted amphibians in a wide range of environmental conditions (21, 60). Past studies have attempted to link *Bd* phenotypic patterns with environmental factors in order to understand how abiotic factors might mitigate or exacerbate disease (5, 30, 43, 61). For example, both Becker et al. (30) and Greener et al. (21) documented considerable phenotypic variation for isolates within the *Bd*GPL that was associated with differential pathogenicity in common susceptible host species (*Lithobates sylvaticus* and *Alytes obstetricans*, respectively). In addition, Lambertini et al. (22) and Muletz-Wolz et al. (31) demonstrated phenotypic variation in morphological characteristics (e.g., zoosporangia size) in multiple isolates from within the *Bd*GPL lineage. However, to date, studies that have tried to link pathogen traits to environmental predictors have not been able to account for the extent of phenotypic variation among *Bd* isolates across different environments [e.g., *Bd* growth has not been linked to any environmental parameters such as mean annual temperature, mean annual precipitation, elevation, etc., (22, 31)].

We predicted that quantifying *Bd* growth and reproductive traits from isolates of the same genotype, but collected across a latitudinal gradient (representing different mean annual air temperature regimes), might show distinct TPCs. We conducted temperature experiments to measure traits related to growth, reproduction, and fitness across the known thermal range of *Bd* and generated TPCs for five isolates from within the *Bd*GPL lineage. Our results reveal informative similarities and differences in several of the measured traits between two genetic lineages (*Bd*GPL1 and *Bd*GPL2) and among five *Bd* isolates.

We found that there was no obvious geographic pattern that could explain the distribution of genetic variants of *Bd*GPL collected across a latitudinal gradient within the United States. Three of our isolates nested within the *Bd*GPL1 clade and each originated from a different latitude (Table 1). Two of the isolates nested within the *Bd*GPL2 clade and similarly originated from different latitudes. Both genetic variants had the same *T*_*opt*_ of 21 °C, but the maximum viability differed between *Bd*GPL1 and *Bd*GPL2. In addition, while both genetic variants had maximum zoospores densities and fecundity at low temperatures (4 °C), there were differences between *Bd*GPL1 and *Bd*GPL2 in these key reproductive traits. These findings corroborate previous studies that suggest considerable variation exists even within a single *Bd* lineage (21, 30). We suggest that there are likely numerous factors contributing to variation within *Bd*GPL in addition to thermal conditions. For example, each isolate for this study was collected from a unique host species (Table 1), with each host species occupying habitats that differ in a multitude of factors, including water pH, drying periods, microbiome composition, and other seasonality effects that likely have a large impact on *Bd* (5, 30, 43). Although it is impractical for *Bd* researchers to eliminate all confounding variables for *Bd* isolate origin, we should nevertheless make efforts to treats isolates identically following isolation (e.g., during laboratory maintenance) and acknowledge these limitations for resolving questions concerning differential pathogenicity.

We found intriguing patterns in the responses of *Bd* to temperature when assessing differences among all five isolates. To begin with, we found that the overall patterns of viability were similar and exhibited a *T*_*opt*_ at the intermediate temperature of 21°C. However, within each temperature, the isolates frequently differed from each other in their maximal viability, zoospore densities, fecundity, growth rates, and carrying capacities. These differences were pronounced at either end of the thermal spectrum, at low (4 °C) and high (26 and 27 °C) temperatures. For example, the temperature of the *T*_*opt*_ for zoospores densities is lower than 21°C, with far more zoospores produced in low temperatures (4 and 12°C), for a subset of the isolates. Furthermore, the fecundity of *Bd* was highest in low temperatures for every isolate. These findings are in line with those from previous studies that suggest understanding *Bd* responses (particularly zoospore production) in low temperatures is important to resolving the complexities of the fundamental niche and the disease ecology of *Bd* (42, 49, 62).

Additionally, we observed interesting patterns of *Bd* viability, growth rates, and carrying capacities at both extremes of the thermal range, making it difficult to determine the true *CT*_*max*_ and *CT*_*min*_. Notably, we found the greatest complexity in thermal responses at the *CT*_*max*_; most of the *Bd* isolates (all except Vermont) exhibited at least some zoosporangia development, and early exponential growth (*r*), in the high temperature treatments (25, 26, and even 27 °C). Yet none of the isolates produced any zoospores and growth could not be sustained for the duration of the experiment. In addition, we found that for the higher temperatures (>21°C) considered in this experiment, the *r* estimates did not decline as one might expect. Rather, it was the *K* estimates that seemed to decline over the upper temperatures. As a result, the *r* estimates (constrained to follow a J–L curve) were overfit to the mid-range temperature data, causing unrealistically high *CT*_*max*_ and *T*_*opt*_ estimates, and poor *r* estimates, for high and low temperatures. Our findings for the higher temperature treatments differ from some previous studies that found no *Bd* growth at temperatures above 24 °C (31, 43, 62). Thus, our findings that *Bd* can remain viable at high temperatures, but fail to produce zoospores, underscore the importance of using a viability assay to investigate additional questions concerning *Bd* responses to temperature (45).

Taken together, the variation in TPCs of the maximum viability of *Bd* isolates collected across a latitudinal gradient did not fit a pattern that could be explained by the “hotter is better” hypothesis; all isolates had the same *T*_*opt*_ for viability at 21°C. Instead, our viability results suggest that a vertical shift model may better explain the patterns for the TPCs of all five isolates. Namely, our viability measurements, and results from carrying capacities (*K*) among isolates, provide some evidence that mean temperatures across latitudes may influence the maximal performance of *Bd*. The isolates from northern latitudes (i.e., Vermont & Ohio), where mean temperatures are generally lower (~4–12 °C; 61), exhibited lower viability and carrying capacities across temperatures, including at their *T*_*opt*_. In contrast, the isolates from Louisiana, New Mexico, and Tennessee, in more southern latitudes where mean temperatures are generally higher (~14–25 °C; 61), exhibited increased viability and carrying capacities across temperatures, including at their *T*_*opt*_. As such, our evidence indicating a vertical shift in TPCs suggest that the mean temperatures experienced by amphibians across a latitudinal gradient may influence maximal viability—but not the *T*_*opt*_ or *CT*_*max*_—of *Bd*. We note, however, that our results for our other reproductive parameters, including zoospore densities and fecundity, did not exhibit a similar pattern, underscoring the importance of measuring multiple traits to gain a full understanding of the complexities of *Bd* responses to temperature (37, 38).

Disease ecologists are concerned with how changes in environmental factors, such as temperature gradients, may influence disease dynamics through alterations in the biology of pathogens such as *Bd* (63, 64). Environmental influences on *Bd* traits such as growth and reproduction may ultimately influence the disease outcomes of chytridiomycosis (42, 44). For example, temperature conditions within local environments may increase viability, zoospores densities, fecundity, growth rates, or carrying capacities of *Bd*, leading to higher infectivity, and greater threat of disease for vulnerable amphibians (49). The threat of biodiversity loss for amphibian communities may be exacerbated from diseases like chytridiomycosis in the coming decades (63). To intervene in the continued population declines of amphibians, we must understand how pathogen biology is mediated across different environments, and within and among genetic lineages. We must also determine what environmental factors are driving the disease dynamics responsible for the disease-induced losses of amphibian biodiversity.

## Data Availability Statement

The data are deposited in the Figshare repository, doi: 10.6084/m9.figshare.14714865, doi: 10.6084/m9.figshare.14714856, and doi: 10.6084/m9.figshare.14714841.

## Author Contributions

CS and JV conceived and executed the study, analyzed results, and wrote the paper. DS and PH conducted several statistical analyses and data visualizations. AB and ER conducted the sequencing and provided genetic information for the isolates. CR-Z provided input on the study design and editorial assistance. All authors contributed to the writing of the manuscript and assisted in the resulting finished product.

## Conflict of Interest

The authors declare that the research was conducted in the absence of any commercial or financial relationships that could be construed as a potential conflict of interest.
