# Supplementary material for: Thermal Performance Curves of Multiple Isolates of Batrachochytrium dendrobatidis, a Lethal Pathogen of Amphibians
Source: Front Vet Sci. 2021 Jun 22;8:687084. doi: 10.3389/fvets.2021.687084 (PMC8258153; doi:10.3389/fvets.2021.687084)
Supplement: Supplementary file 1 [file Data_Sheet_1.PDF]

Supplementary Material

The following document provides additional details on the logistic curve fitting and comparisons across isolates and strains.

Fitting logistic growth curves

1. For a single isolate at a single temperature, we fit a logistic curve to the MTTn data. The model is a standard logistic growth curve with parameters x_0, r, and K: a solution to the differential equation

$$\frac{dX}{dt} = r\,X\,(1 - X/K)$$

   that starts at $X(0) = x_0$. The data are assumed to be described by this model as follows: This curve, at time point t_i, defines the mean of a Normal distribution $\mu_i = x(t_i)$ with standard deviation σ_i that is a positive linear function of the mean, specifically, $\sigma_i = b_0 + b_1\,\mu_i$, where all paramters are non-negative (to avoid running into the numberical issue where $\sigma_i = 0$ we have below imposed a minimum value to require that $b_0 \geq 10^{-4}$).

2. To fit this model to each time series, we use maximum likelihood estimation for the model described above, and use the optim() function in R to find the parameter set that yields the minimum negative log likelihood. To ensure this optimization procedure converges properly, we use the multi-start approach where we repeat this optimization step $N >> 1$ times, each time starting at a different initial point in parameter space. We then use the best of these N estimates.

Likelihood Profiling: 95% Confidence intervals for r and K

Here we use likelihood profiles to obtain approximate 95% confidence intervals for parameters r and K. This is done as follows.

For the parameter of interest (call it θ), we take the best fit parameter set (with $\theta = \theta_0$) and consider a range of values over an interval that extends above and below θ_0. For each of those fixed values of $\theta \neq \theta_0$ (let's refer to one such fixed value with the notation θ_*), we re-run the parameter estimation procedure to re-fit the model where $\theta = \theta_*$ is fixed, and the optimization procedure can vary all of the other parameters. This yields a best fit parameter given the constraint that $\theta = \theta_*$, and we can do a likelihood ratio test to compare the goodness of fit between the best fit ($\theta = \theta_0$) model parameterization and this alternative model parameterization (with $\theta = \theta_*$ and one less degree of freedom).

The test statistic

$$D = -2\,(nLL_0 - nLL_*)$$

is asymptotically χ^2 distributed with 1 degree of freedom. Therefore we can test whether this alternative parameterization is significally different from the best fit model, and this allows us to compute a confidence interval by repeating this procedure for a range of θ values above and below θ_0, then plotting the test statistic D as a function of these different θ_* values. For a significance threshold at the $p \times 100\%$ confidence level, any models for which the likelihood ratio test statistic D is below $D_p =$ `qchisq(p=p,df=1)` are considered not significantly different from the best fit model. Here we have selected a 95% confidence threshold (`qchisq(p=0.95,df=1)`) which is approximately 3.48, shown as a red dashed line on the graphs below, however we also show a 99% threshold value of approximately 6.63 (those CIs are not reported).

Lastly, because we only consider a finite range of parameter values above and below the best-fit parameter value, in some cases we were unable to detect the upper limit for the CI due to apparent *identifiability* issues (e.g., where x_0 is trading-off with r or where exponential-phase data are fit equally well by logistic growth curves with arbitrarily large K values). Plots for such cases have CIs plotted with dotted lines, rather than solid lines.

Plots and Associated Outputs

Logistic growth curve fitting

```
## [1] "Parameter Estimates: "
##    isolate Temp           x0         r            K           b0           b1
## 1       LA    4 1.078206e-03 0.2590634 6.245595e-01 2.191184e-03 2.844694e-01
## 2       LA   12 7.672062e-03 0.5867231 8.337093e-01 6.476346e-03 1.616338e-01
## 3       LA   17 8.383161e-03 0.8539170 1.276013e+00 1.163975e-02 1.643746e-01
## 4       LA   21 5.453235e-03 1.5042143 1.047551e+00 7.896498e-03 2.560711e-01
## 5       LA   25 9.907636e-03 0.9155305 9.878376e-01 5.099038e-03 1.015925e-01
## 6       LA   26 1.672806e-02 0.4820032 1.417591e+06 1.610885e-02 1.997543e-01
## 7       LA   27 3.298714e-04 2.9465853 2.611439e-02 3.484856e-13 8.336986e-01
## 8       NM    4 7.017758e-04 0.1938702 1.047245e-01 3.437669e-03 2.776075e-01
## 9       NM   12 7.763511e-04 0.6296962 5.608233e-01 6.427791e-03 1.964012e-01
## 10      NM   17 1.569133e-02 0.8780131 8.240407e-01 1.190991e-02 1.799626e-01
## 11      NM   21 2.659761e-02 0.8080043 8.820931e-01 2.780935e-02 8.082133e-02
## 12      NM   25 7.644358e-04 1.3242149 4.455282e-01 2.640026e-03 1.247623e-01
## 13      NM   26 1.623522e-03 1.1802665 2.762338e-01 4.793743e-11 3.149722e-01
## 14      NM   27 1.457109e-03 1.0628639 7.503278e-02 7.448617e-04 3.302224e-01
## 15      OH    4 2.368872e-04 0.1925829 6.490205e-02 5.060213e-03 2.208802e-01
## 16      OH   12 3.522725e-03 0.3071427 2.312914e-01 4.529568e-03 2.397883e-01
## 17      OH   17 1.744314e-04 0.9106411 5.444790e-01 6.236108e-03 1.046281e-01
## 18      OH   21 1.420256e-03 0.8263378 6.487234e-01 5.343165e-03 1.851403e-01
## 19      OH   25 2.184343e-03 1.5214743 3.902704e-01 2.821321e-05 2.776008e-01
## 20      OH   26 1.868559e-03 1.4512178 2.993768e-01 1.253385e-03 3.462211e-01
## 21      OH   27 3.171476e-03 1.0015790 1.116322e-01 3.060500e-12 3.895854e-01
## 22      TN    4 1.911496e-04 0.1832258 2.250046e-02 4.518278e-03 1.836797e-01
## 23      TN   12 2.471917e-04 0.8495657 4.357259e-01 1.269300e-02 1.152454e-01
## 24      TN   17 4.291135e-04 0.7361102 6.317436e-01 4.631531e-03 1.075680e-01
## 25      TN   21 1.614565e-03 0.8276446 6.701784e-01 6.009578e-03 2.218679e-01
## 26      TN   25 2.319097e-03 1.0170940 3.259483e-01 2.191698e-03 1.932003e-01
## 27      TN   26 1.509779e-03 1.2371524 1.559253e-01 1.060328e-03 4.333080e-01
## 28      TN   27 2.421457e-04 1.8740154 5.331168e-02 3.883148e-03 2.502553e-01
## 29      VT    4 8.794540e-05 0.1744660 5.413685e-01 4.315316e-03 2.000063e-01
## 30      VT   12 4.056181e-03 0.2312362 4.757365e-01 6.644453e-03 1.262364e-01
## 31      VT   17 6.070686e-03 0.4320938 6.248107e-01 6.377395e-03 1.346735e-01
## 32      VT   21 1.689098e-02 0.3991459 6.020393e-01 1.619324e-02 9.052296e-02
## 33      VT   25 2.177900e-04 0.9505612 7.155160e-02 3.640874e-03 1.022782e-01
## 34      VT   26 2.719409e-18 8.5830803 1.922429e-02 2.235604e-03 4.546393e-01
## 35      VT   27 2.217344e-06 1.1167202 4.244246e+01 3.122041e-03 3.359615e-10
##    convcode        NLL nobs
## 1         0 -161.81533   75
## 2         0  -79.21697   80
## 3         0  -41.35193   38
## 4         0  -24.35651   38
## 5         0  -73.27635   35
## 6         0  -59.99346   35
## 7         0  -80.68266   30
## 8         0 -236.96651   75
## 9         0 -110.41979   80
## 10        0  -40.24652   34
## 11        0  -45.60532   35
## 12        0  -98.14405   35
## 13        0  -85.63105   35
## 14        0 -109.22662   35
## 15        0 -254.02903   75
## 16        0 -167.73740   80
## 17        0 -102.95198   40
## 18        0  -77.37027   40
## 19        0  -64.93115   35
## 20        0  -64.38445   35
## 21        0  -90.44952   35
## 22        0 -274.69497   74
## 23        0 -135.04664   80
## 24        0 -109.88249   40
```

```
## 25        0  -69.06880   40
## 26        0  -92.35807   35
## 27        0  -79.81859   35
## 28        0  -94.91163   32
## 29        0 -274.12872   75
## 30        0 -187.27877   80
## 31        0  -96.00241   40
## 32        0  -78.68276   40
## 33        0 -134.44692   35
## 34        0 -137.31954   35
## 35        0 -151.15800   35
```

Individual time series plots with best fit logistic curves

Time series for each isolate at each temperature are shown. The first and second panels are the normalized MTT (MTTn) time series with the overlaid best fit logistic curve. The second panel uses fixed axes so that different time series can be more easily compared visually. The third panel is just the unnormalized OD values, for a reference.

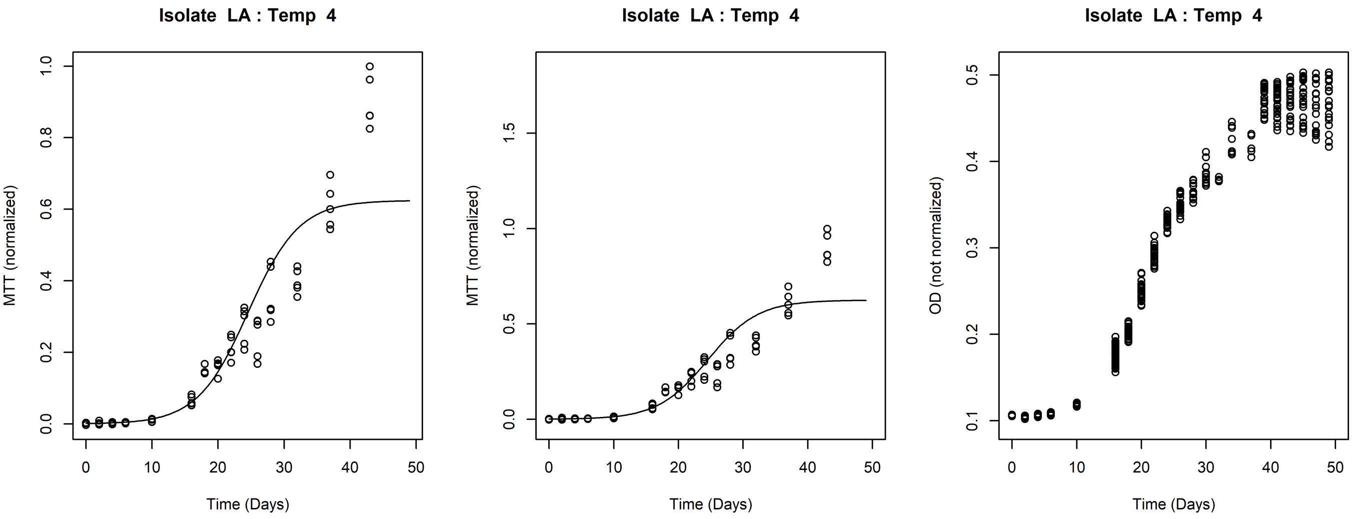


```
##          x0      r      K       b0     b1 convcode
## 1 0.001078 0.2591 0.6246 0.002191 0.2845        0
```

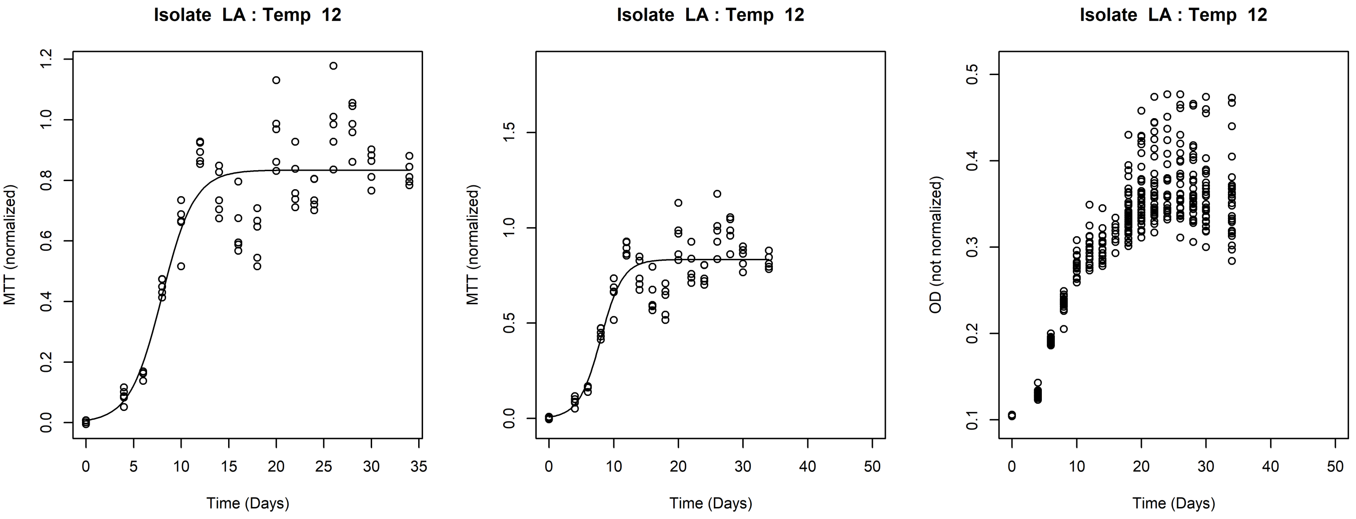

```
##          x0      r      K       b0     b1 convcode
## 2 0.007672 0.5867 0.8337 0.006476 0.1616        0
```

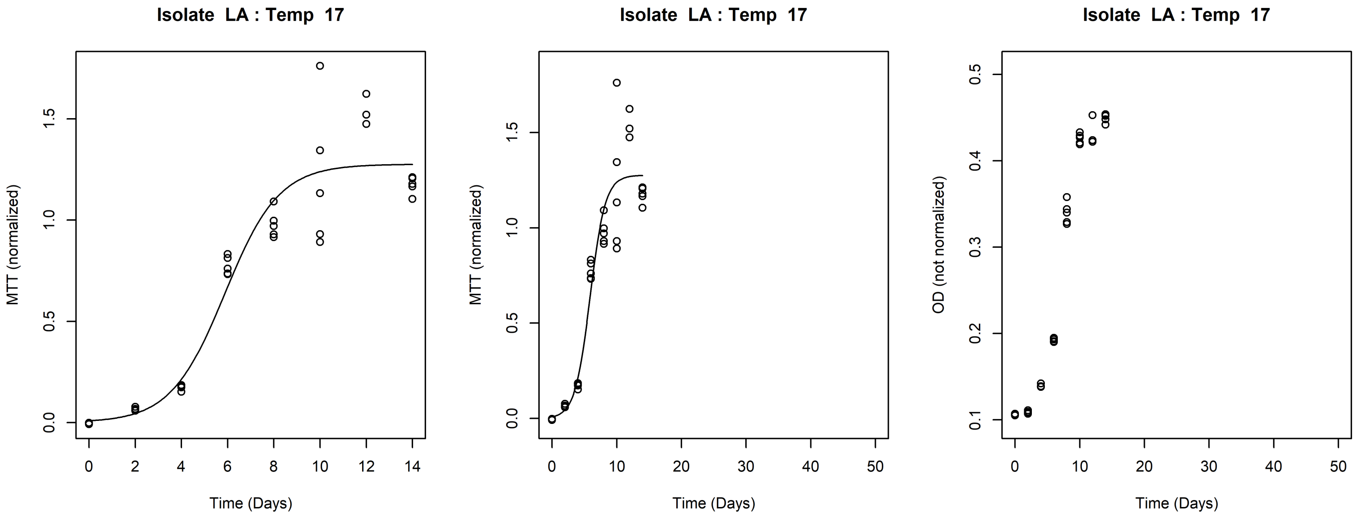


```
##          x0      r     K      b0     b1 convcode
## 3 0.008383 0.8539 1.276 0.01164 0.1644        0
```

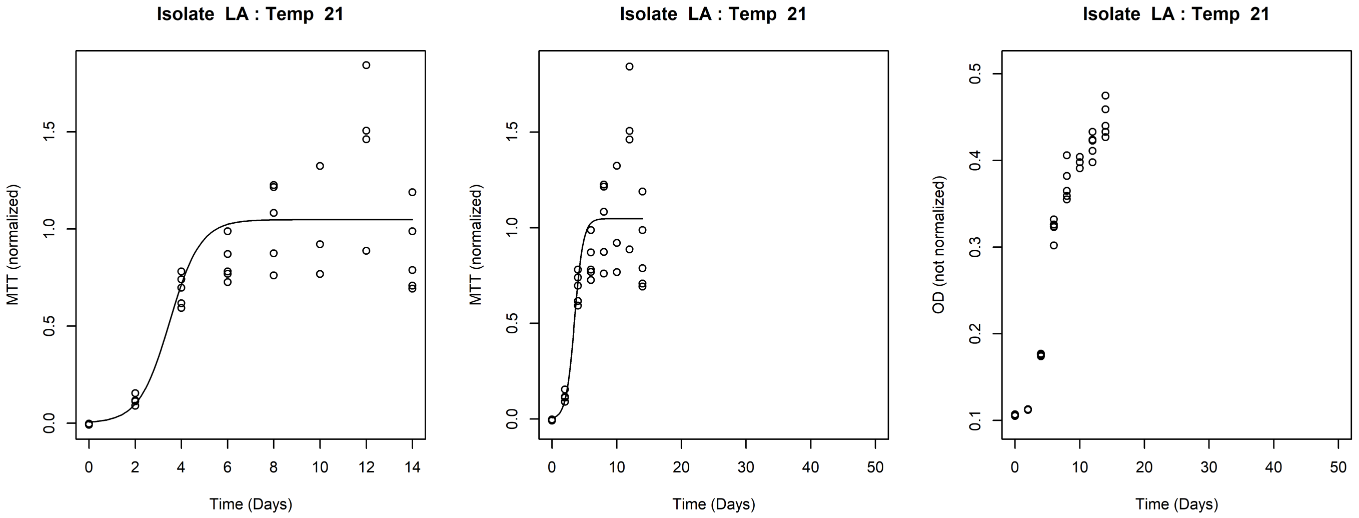


```
##          x0     r     K       b0     b1 convcode
## 4 0.005453 1.504 1.048 0.007896 0.2561        0
```

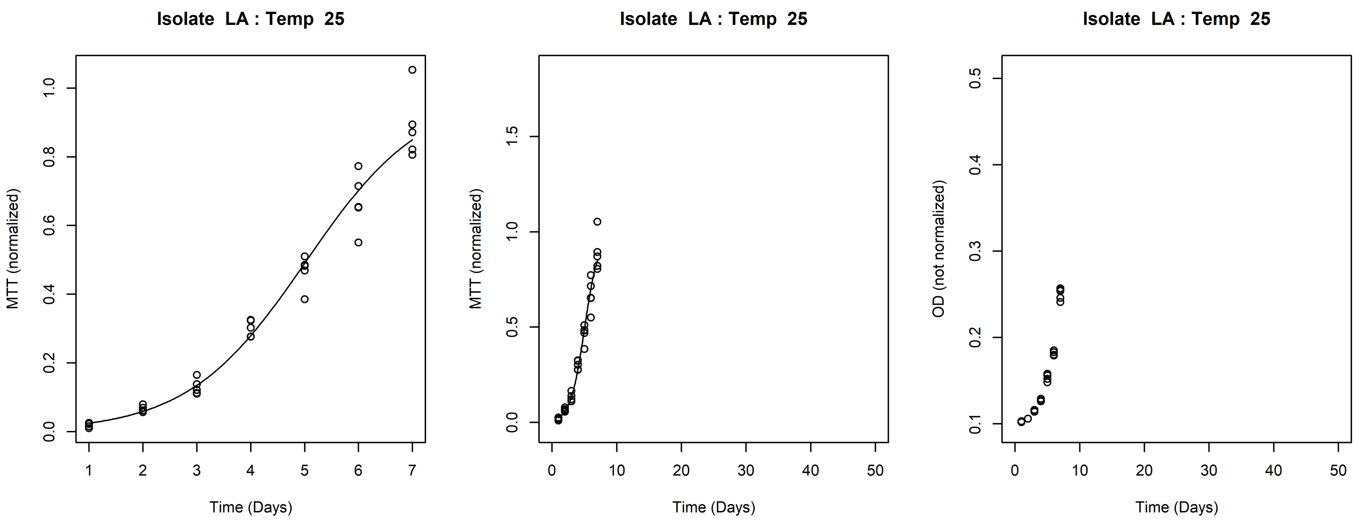


```
##         x0      r      K       b0     b1 convcode
## 5 0.009908 0.9155 0.9878 0.005099 0.1016        0
```

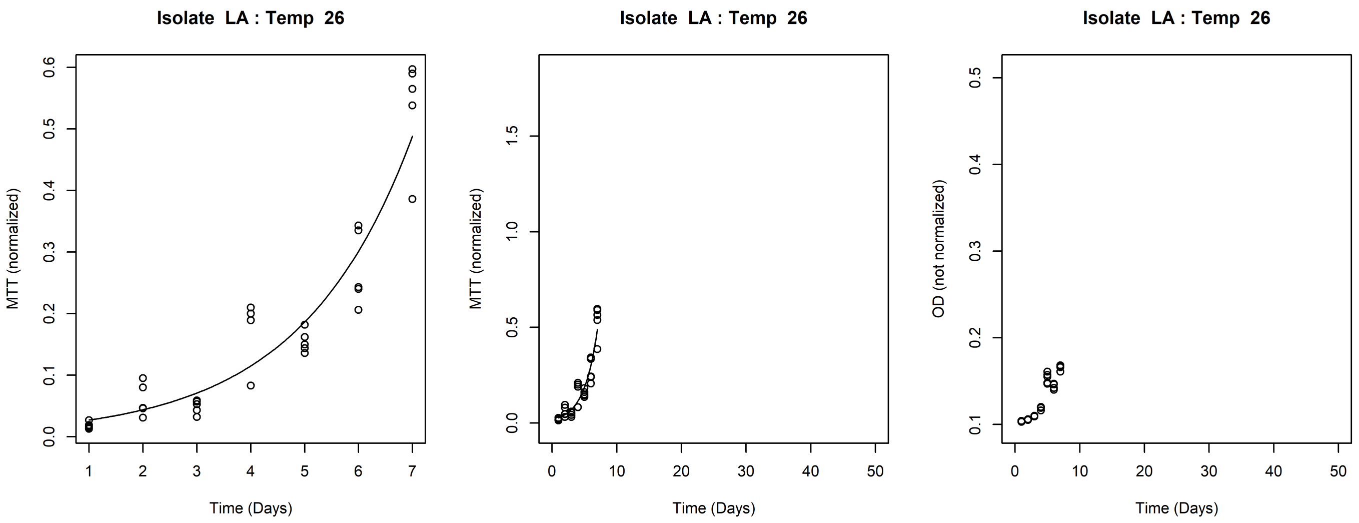


```
##        x0     r       K      b0     b1 convcode
## 6 0.01673 0.482 1418000 0.01611 0.1998        0
```

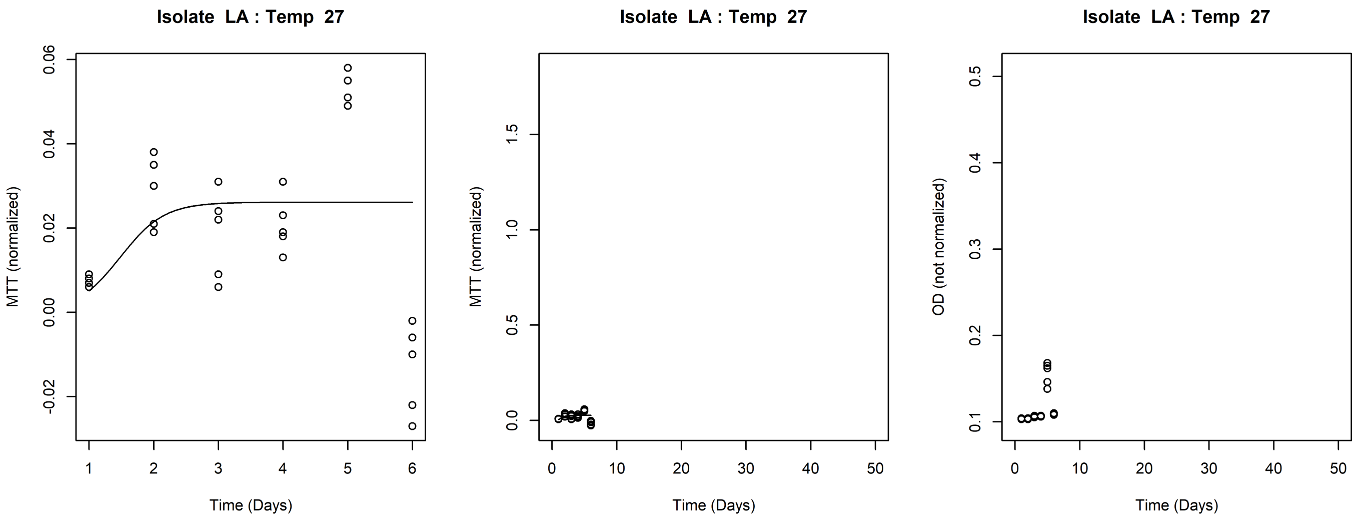

```
##           x0     r       K         b0     b1 convcode
## 7 0.0003299 2.947 0.02611 3.485e-13 0.8337        0
```

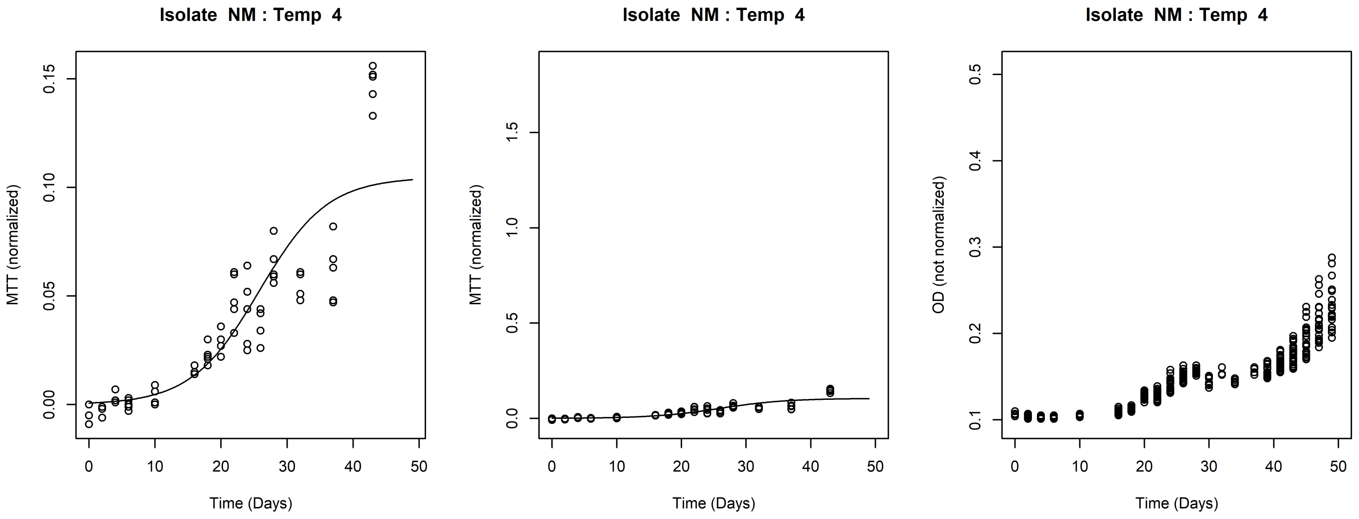


```
##           x0      r      K        b0     b1 convcode
## 8 0.0007018 0.1939 0.1047 0.003438 0.2776        0
```

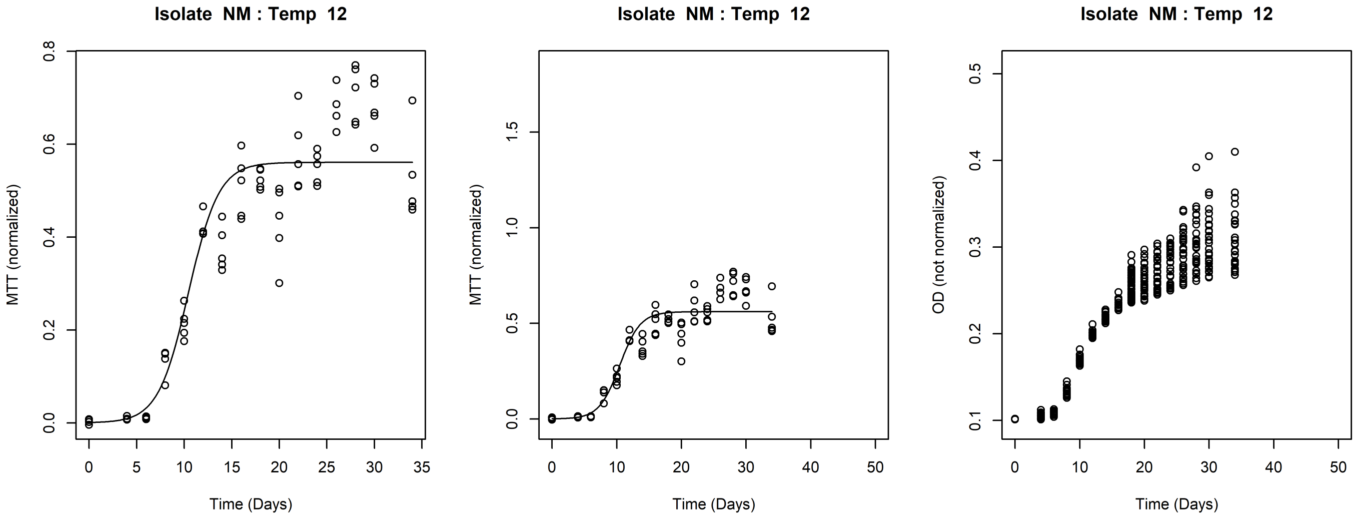


```
##           x0      r      K        b0     b1 convcode
## 9 0.0007764 0.6297 0.5608 0.006428 0.1964        0
```

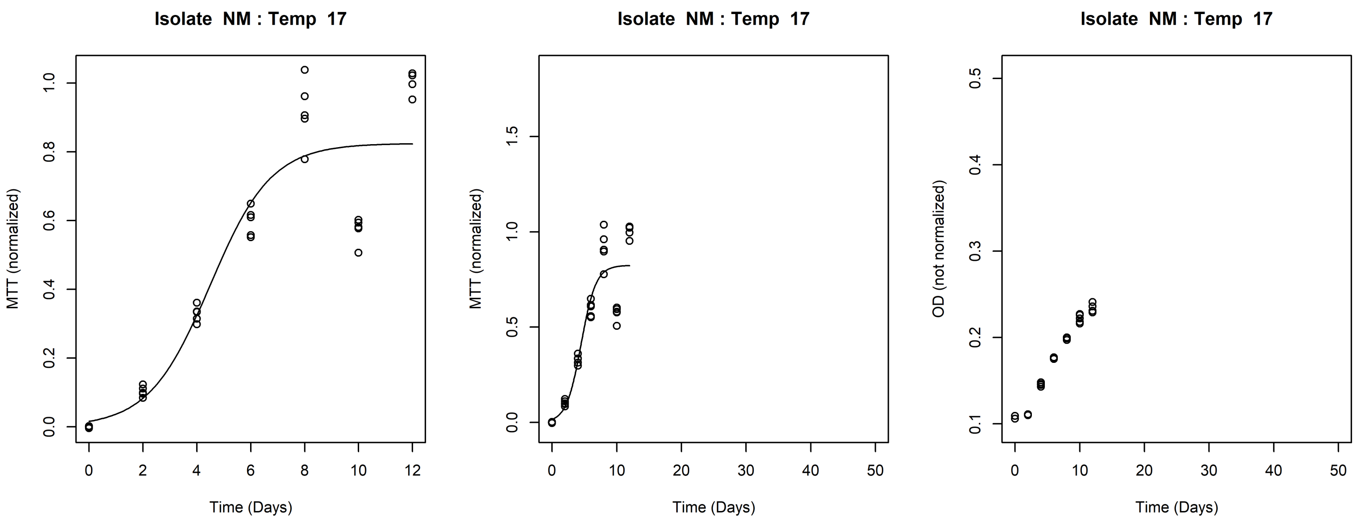


```
##         x0     r     K      b0   b1 convcode
## 10 0.01569 0.878 0.824 0.01191 0.18        0
```

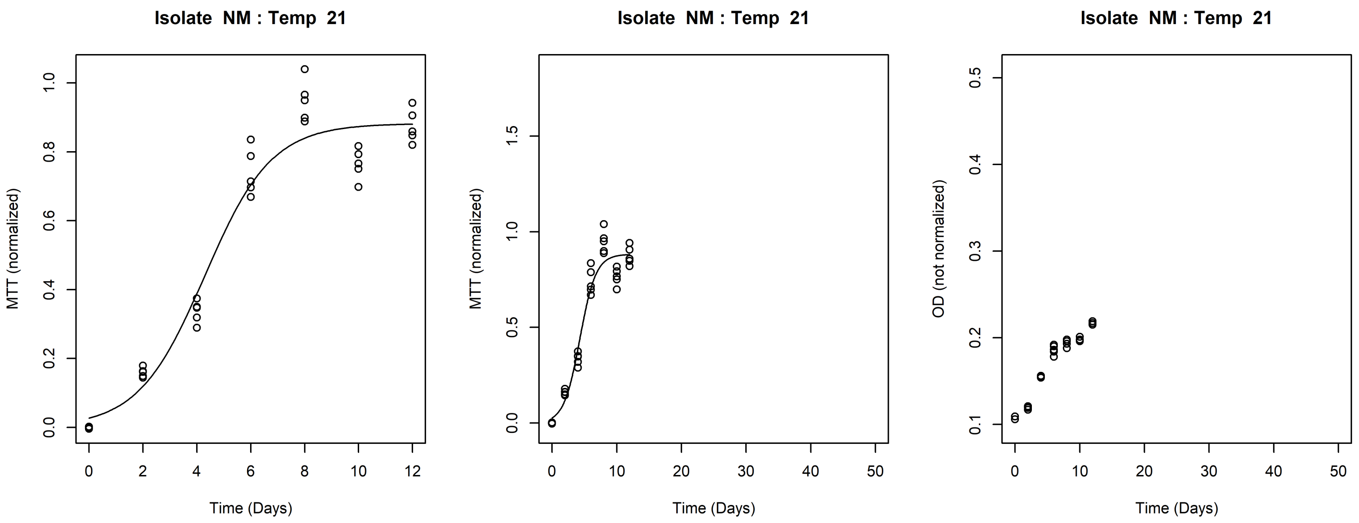


```
##        x0     r      K      b0      b1 convcode
## 11 0.0266 0.808 0.8821 0.02781 0.08082        0
```

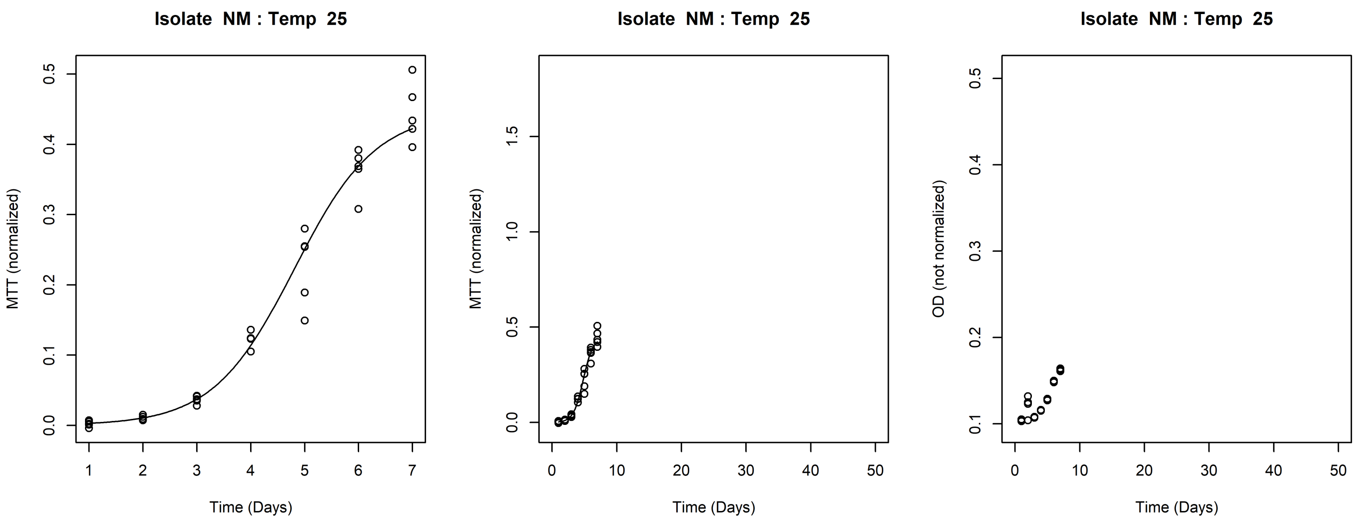

```
##           x0     r      K      b0     b1 convcode
## 12 0.0007644 1.324 0.4455 0.00264 0.1248        0
```

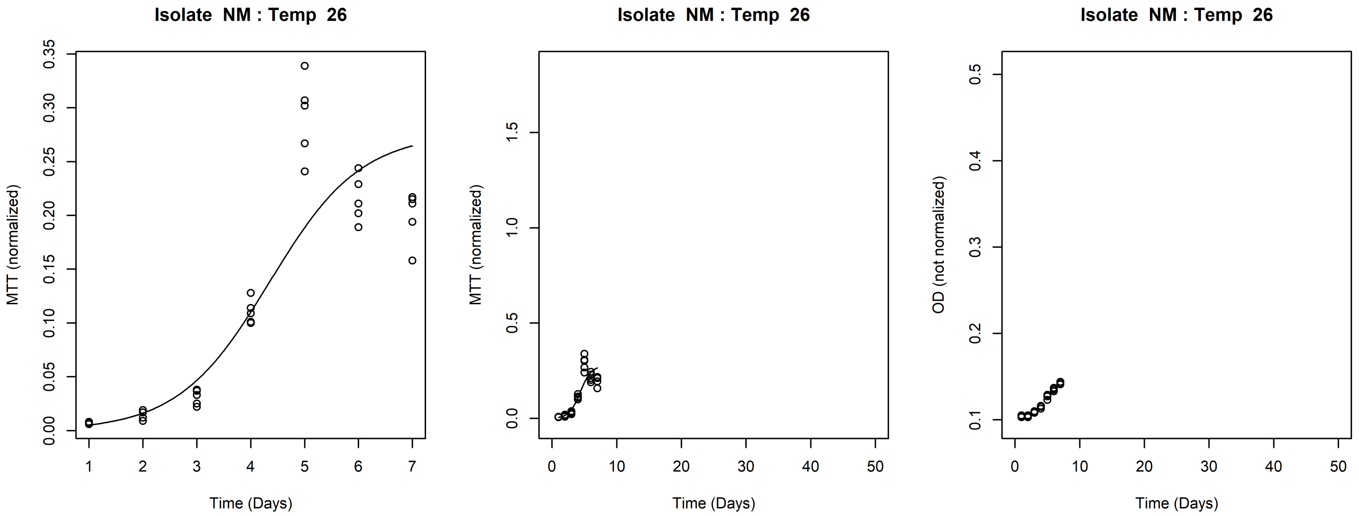


```
##          x0    r      K        b0    b1 convcode
## 13 0.001624 1.18 0.2762 4.794e-11 0.315        0
```

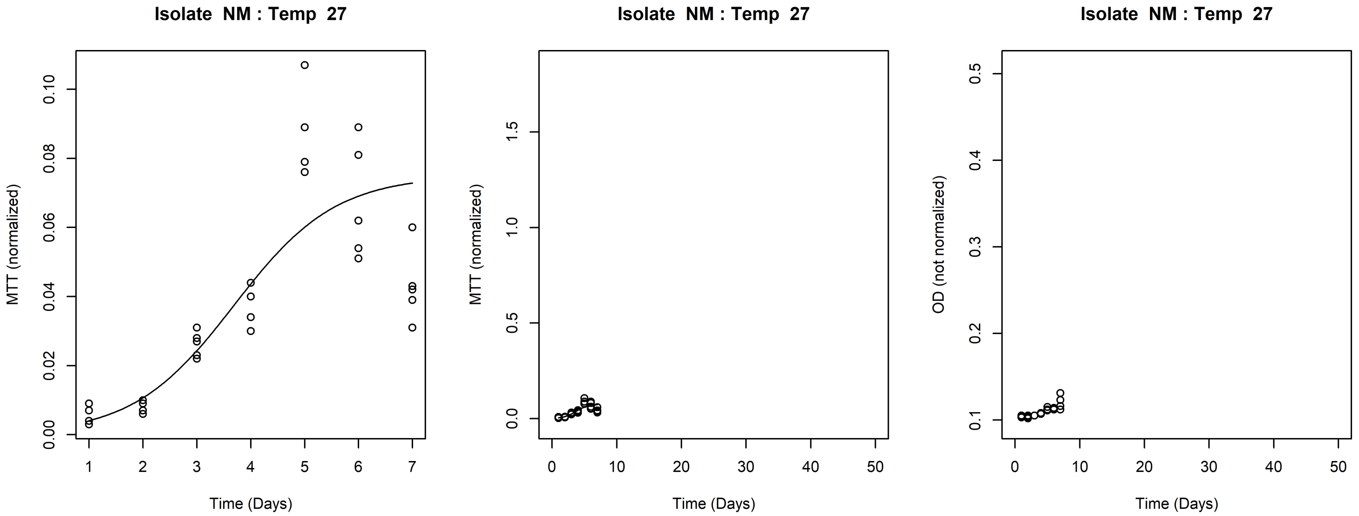


```
##          x0     r       K        b0     b1 convcode
## 14 0.001457 1.063 0.07503 0.0007449 0.3302        0
```

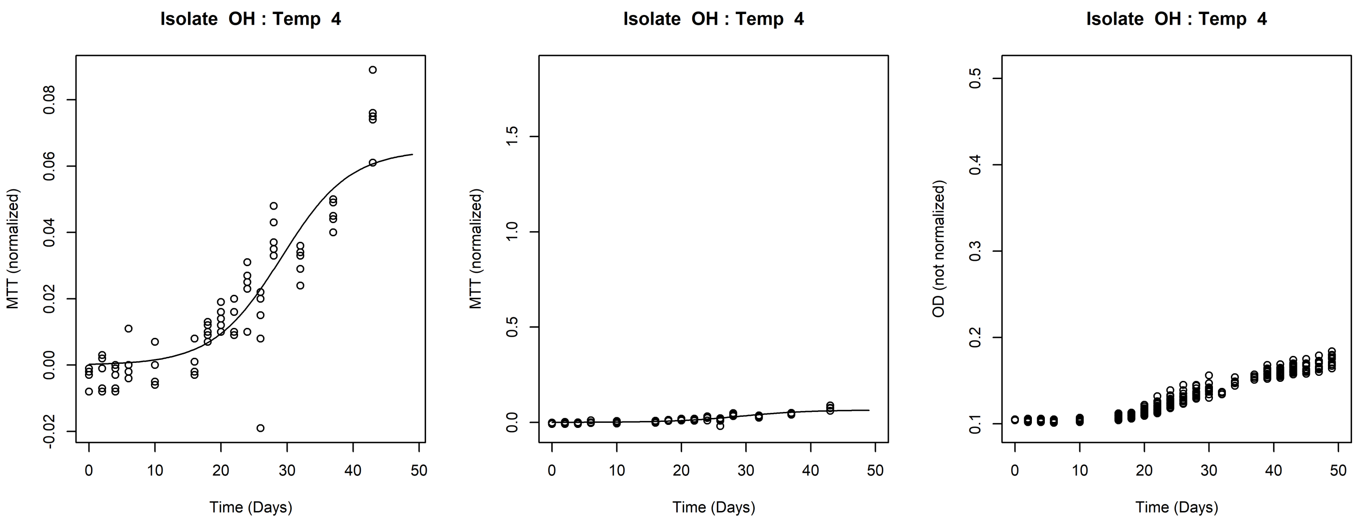


```
##          x0      r      K      b0     b1 convcode
## 15 0.0002369 0.1926 0.0649 0.00506 0.2209        0
```

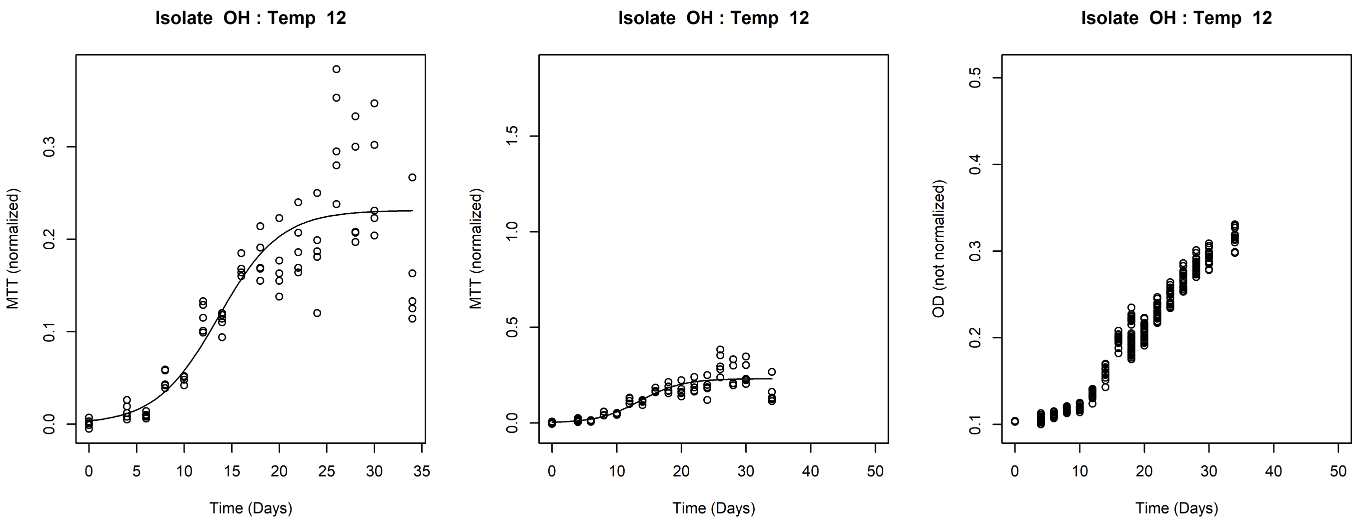


```
##         x0      r      K      b0     b1 convcode
## 16 0.003523 0.3071 0.2313 0.00453 0.2398        0
```

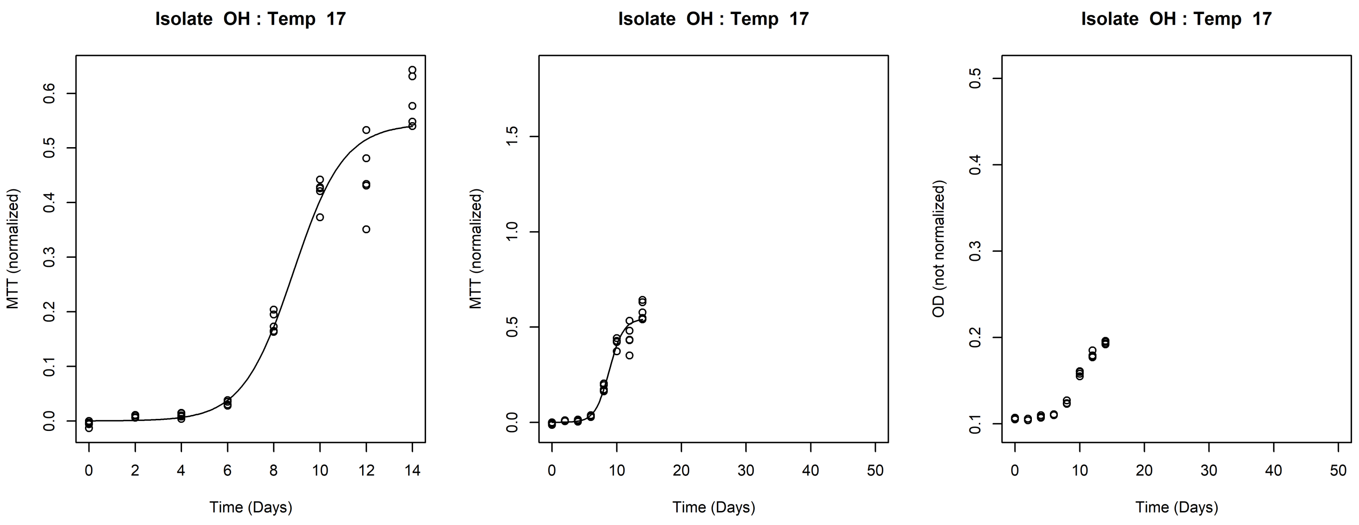

```
##           x0      r      K        b0     b1 convcode
## 17 0.0001744 0.9106 0.5445 0.006236 0.1046        0
```

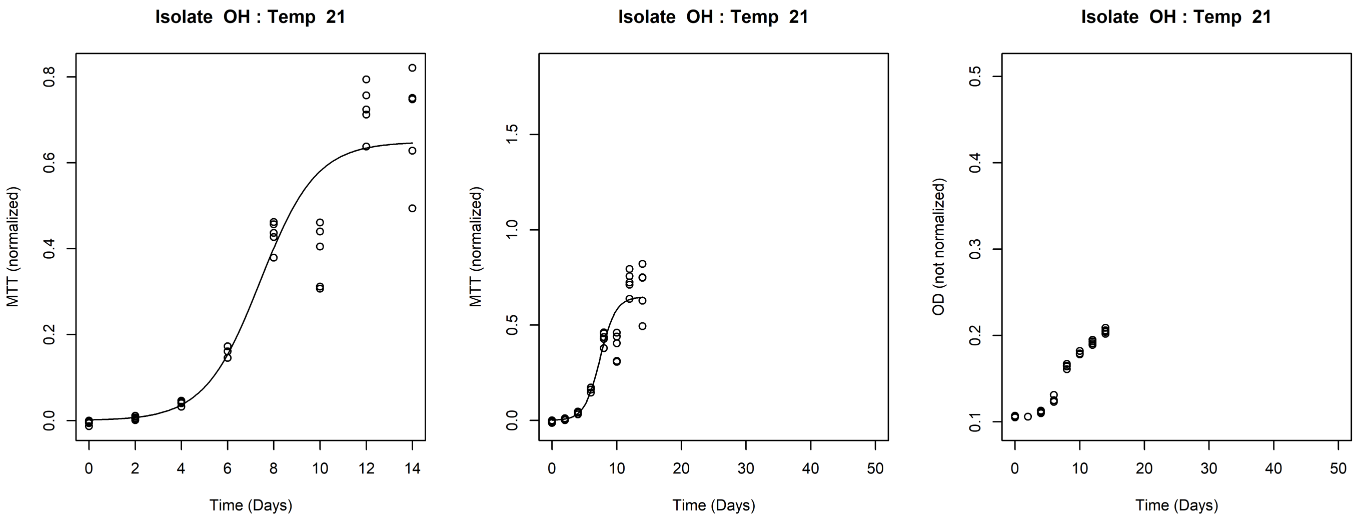


```
##         x0      r      K        b0     b1 convcode
## 18 0.00142 0.8263 0.6487 0.005343 0.1851        0
```

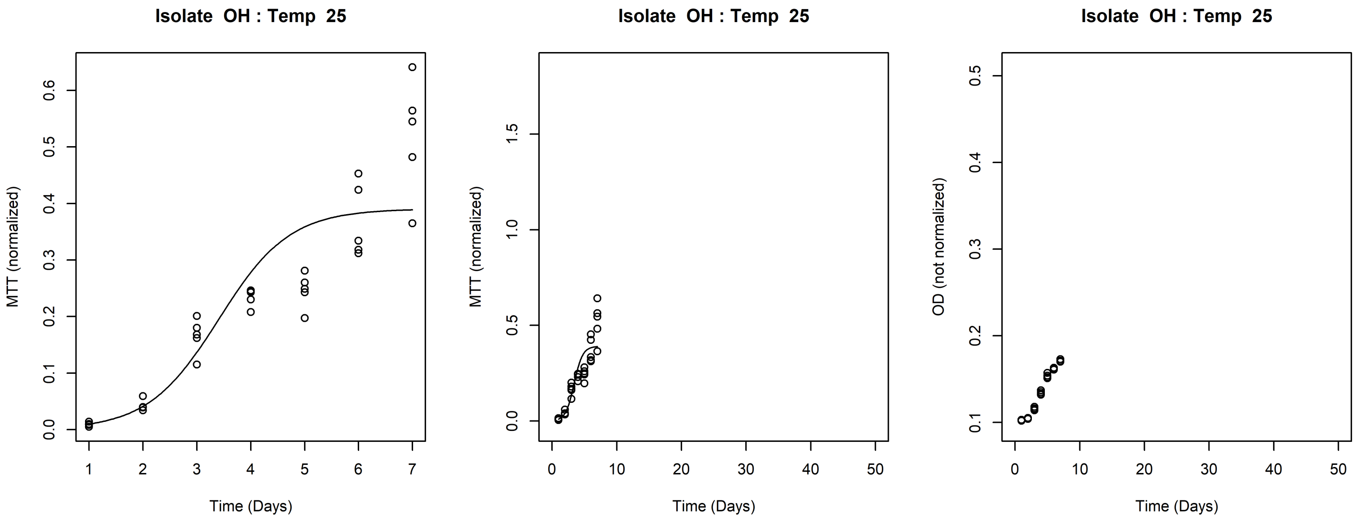


```
##          x0     r      K         b0     b1 convcode
## 19 0.002184 1.521 0.3903 2.821e-05 0.2776        0
```

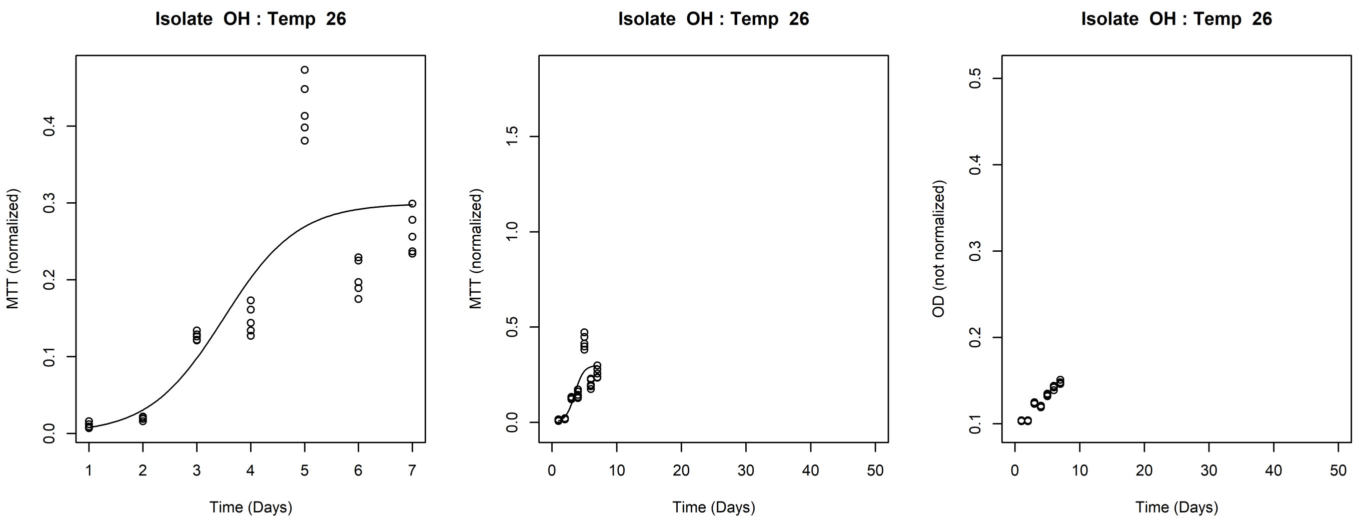


```
##          x0     r      K       b0     b1 convcode
## 20 0.001869 1.451 0.2994 0.001253 0.3462        0
```

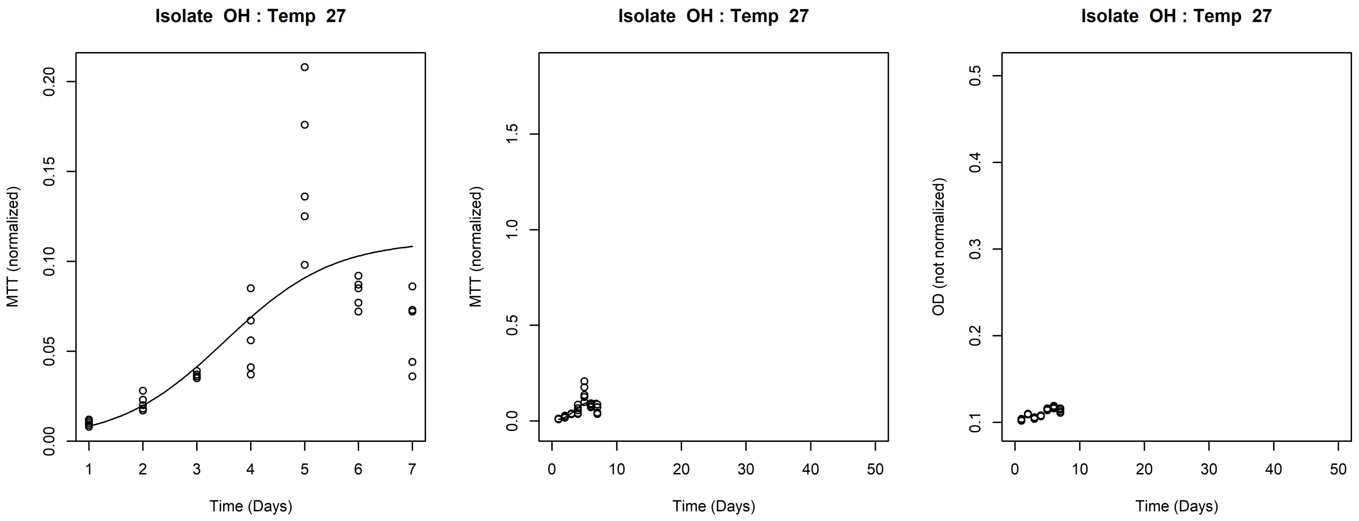


```
##          x0     r      K       b0     b1 convcode
## 21 0.003171 1.002 0.1116 3.06e-12 0.3896        0
```

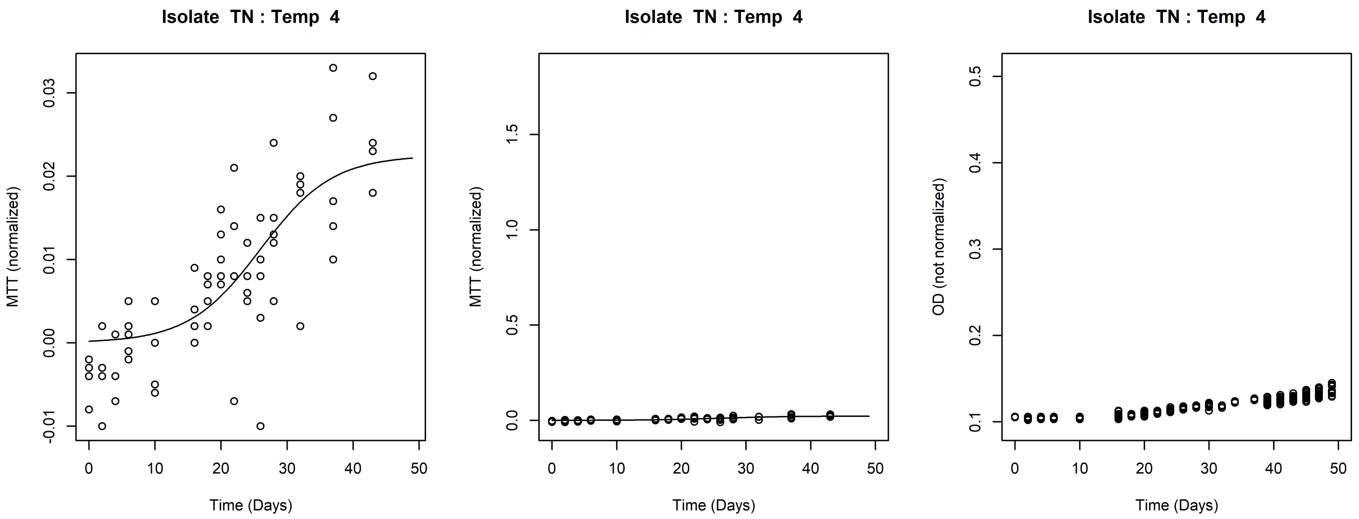

```
##            x0      r      K       b0     b1 convcode
## 22 0.0001911 0.1832 0.0225 0.004518 0.1837        0
```

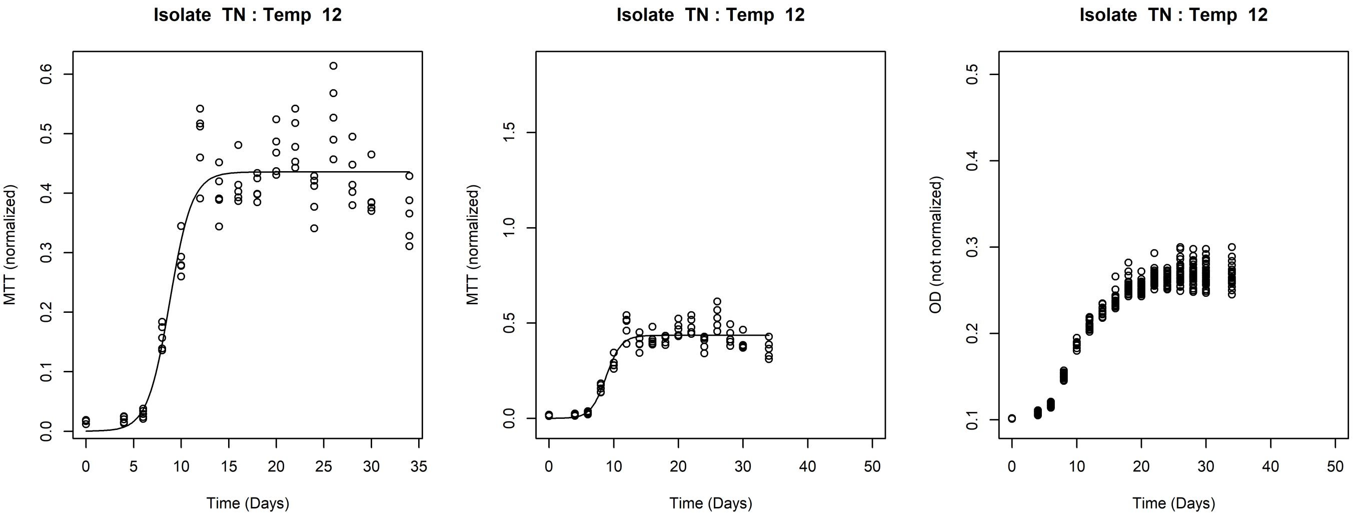


```
##            x0      r      K       b0     b1 convcode
## 23 0.0002472 0.8496 0.4357 0.01269 0.1152        0
```

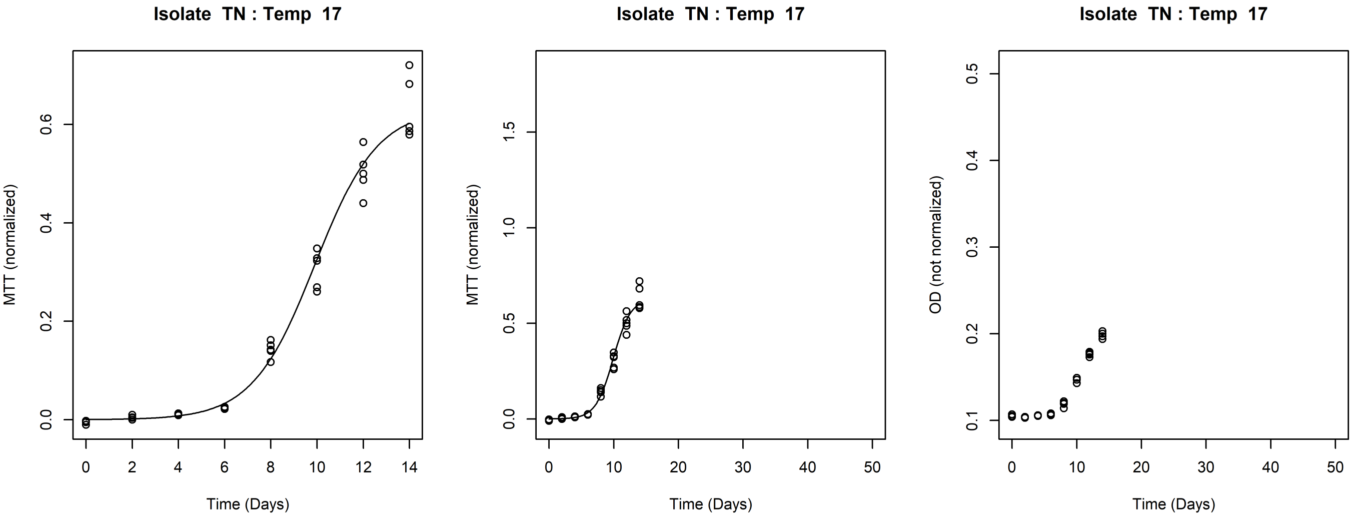


```
##            x0      r      K       b0     b1 convcode
## 24 0.0004291 0.7361 0.6317 0.004632 0.1076        0
```

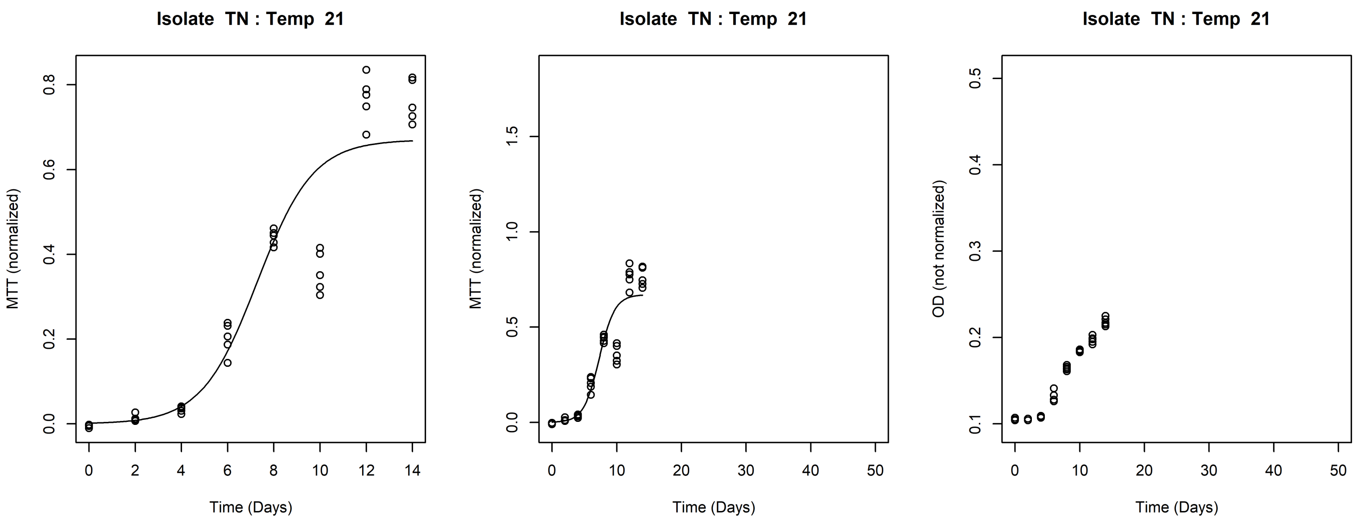


```
##          x0      r      K      b0     b1 convcode
## 25 0.001615 0.8276 0.6702 0.00601 0.2219        0
```

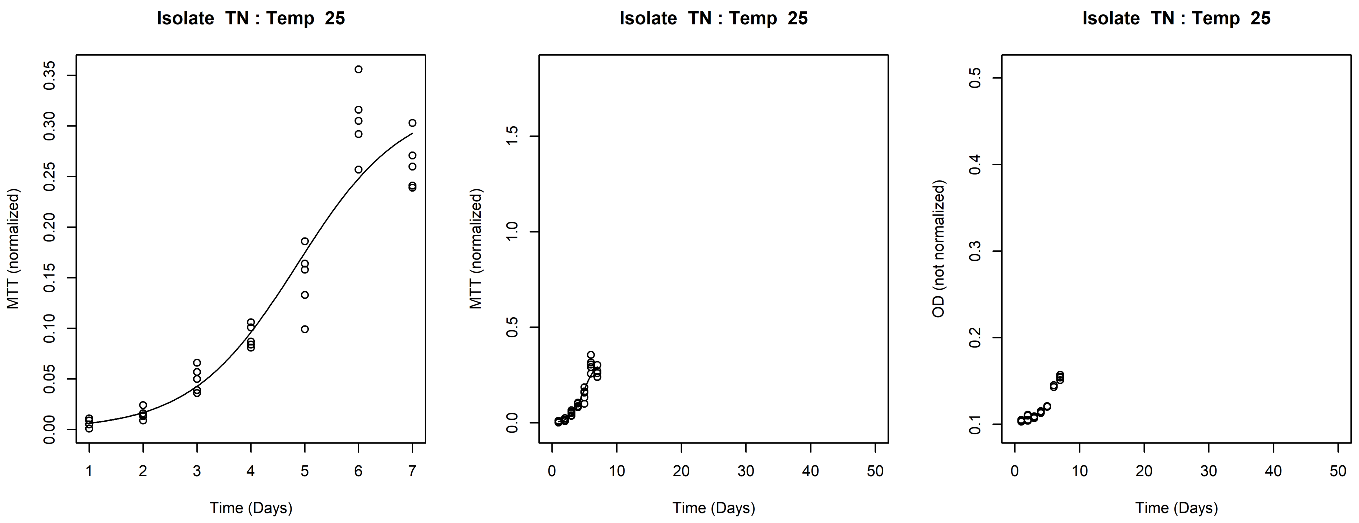


```
##          x0     r      K       b0     b1 convcode
## 26 0.002319 1.017 0.3259 0.002192 0.1932        0
```

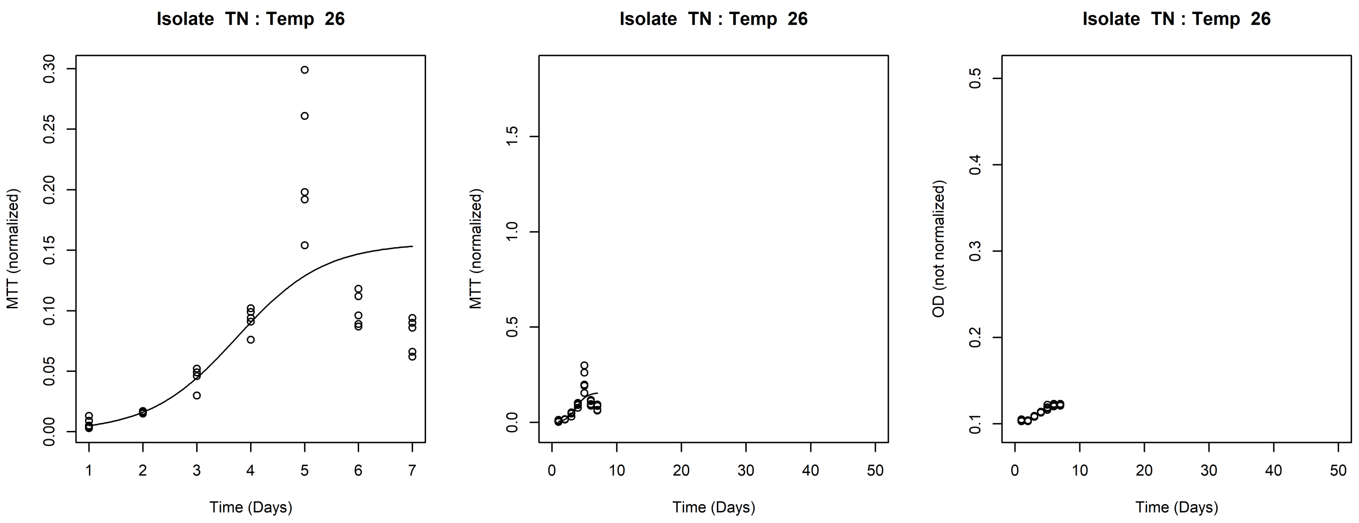

```
##          x0     r      K      b0     b1 convcode
## 27 0.00151 1.237 0.1559 0.00106 0.4333        0
```

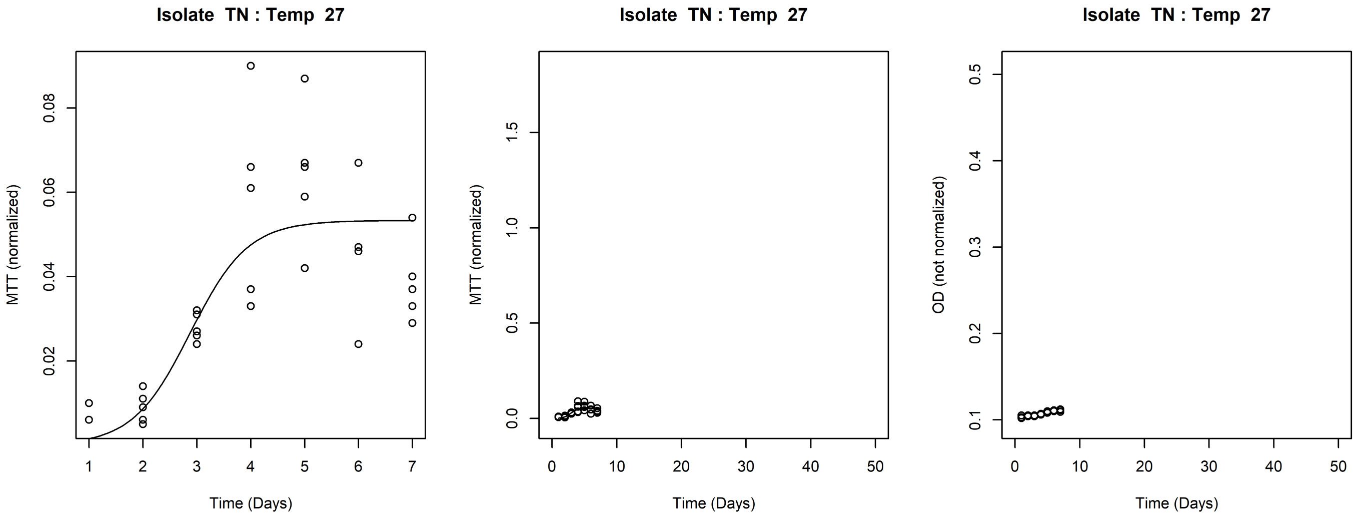


```
##            x0     r       K       b0     b1 convcode
## 28 0.0002421 1.874 0.05331 0.003883 0.2503        0
```

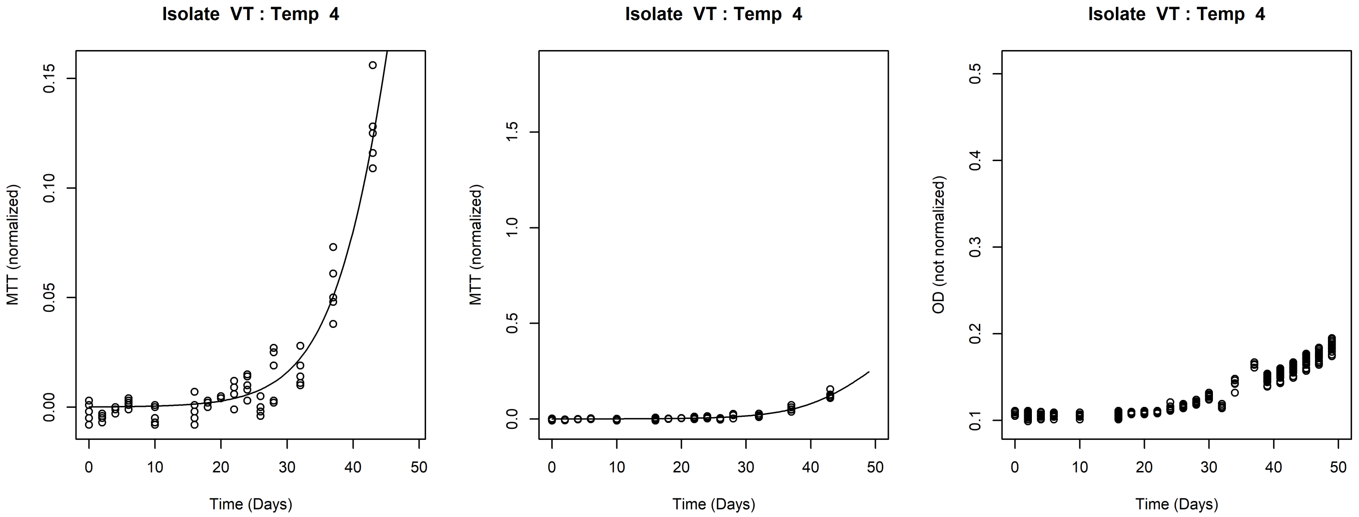


```
##            x0      r      K       b0  b1 convcode
## 29 8.795e-05 0.1745 0.5414 0.004315 0.2        0
```

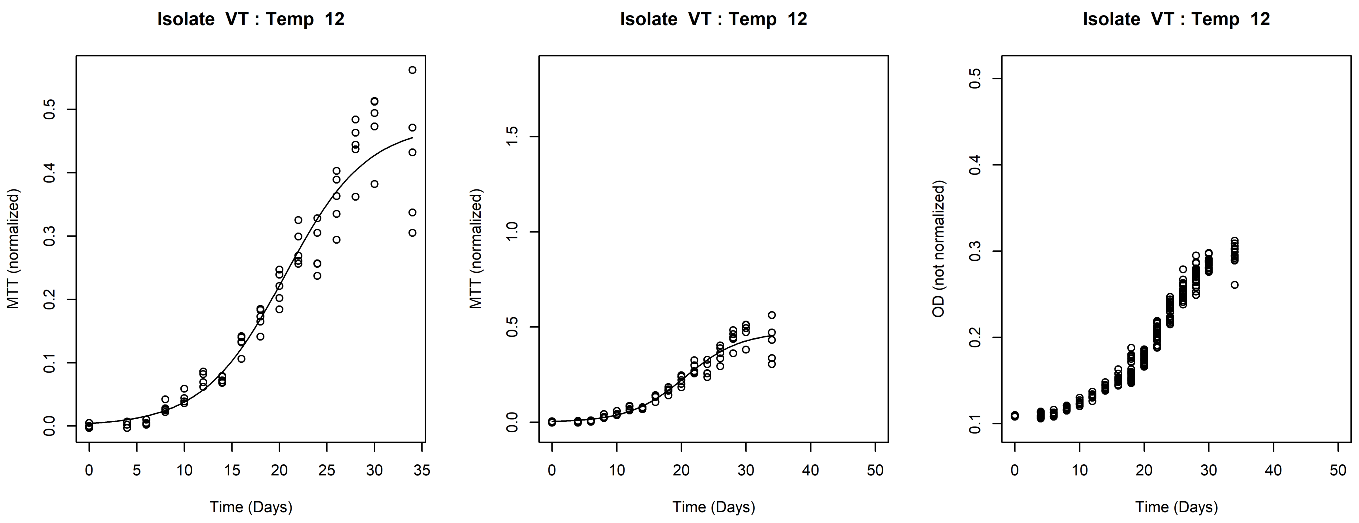


```
##          x0      r      K       b0     b1 convcode
## 30 0.004056 0.2312 0.4757 0.006644 0.1262        0
```

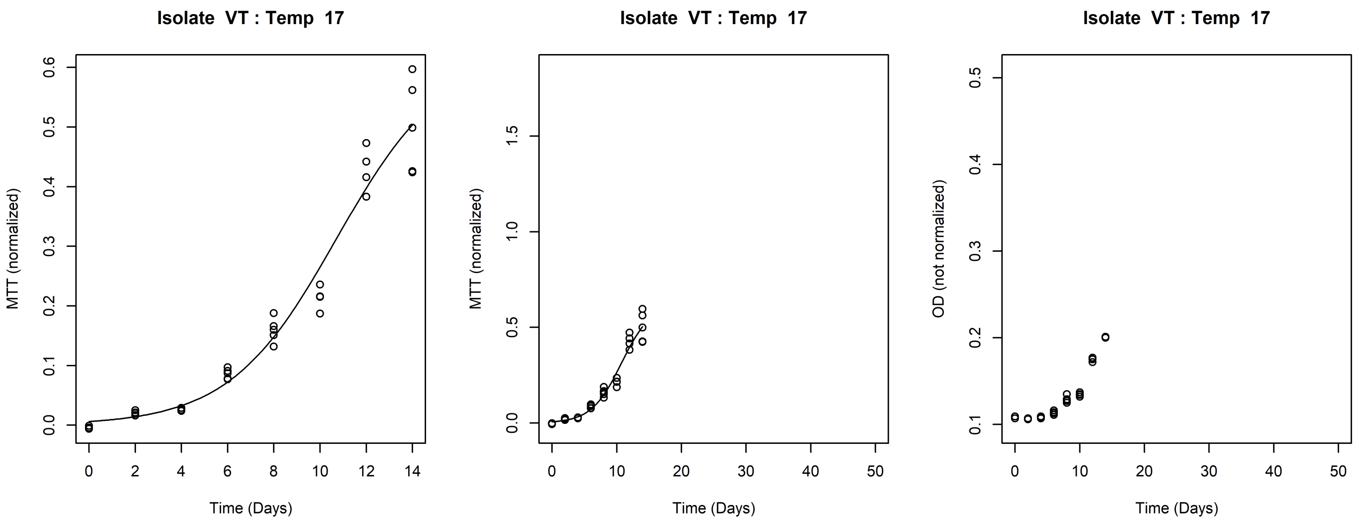


```
##          x0      r      K       b0     b1 convcode
## 31 0.006071 0.4321 0.6248 0.006377 0.1347        0
```

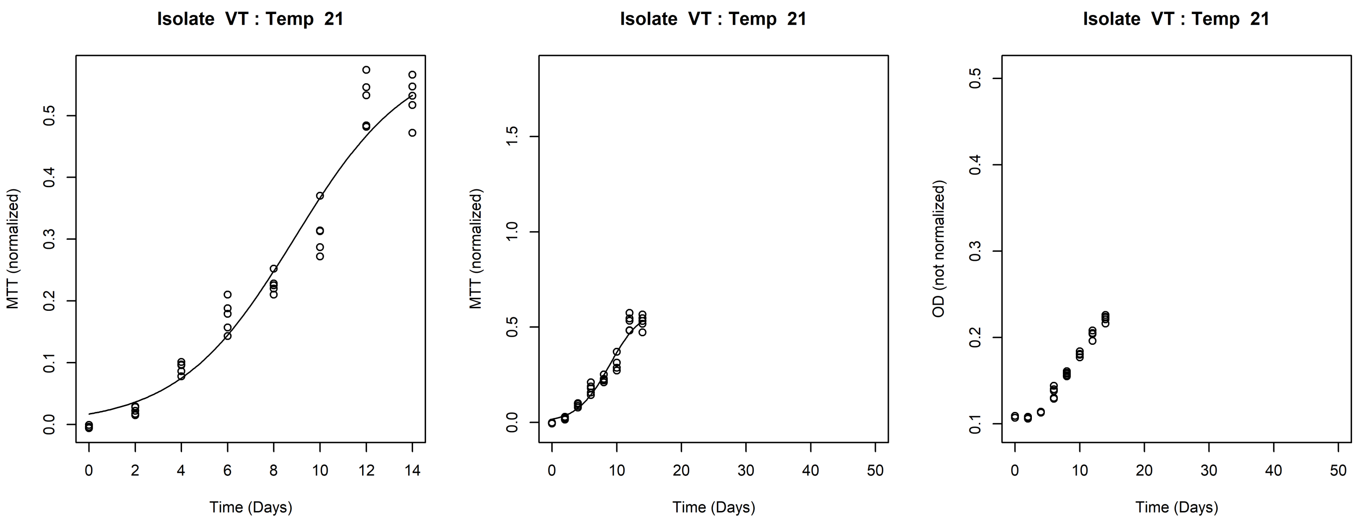

```
##         x0      r     K      b0      b1 convcode
## 32 0.01689 0.3991 0.602 0.01619 0.09052        0
```

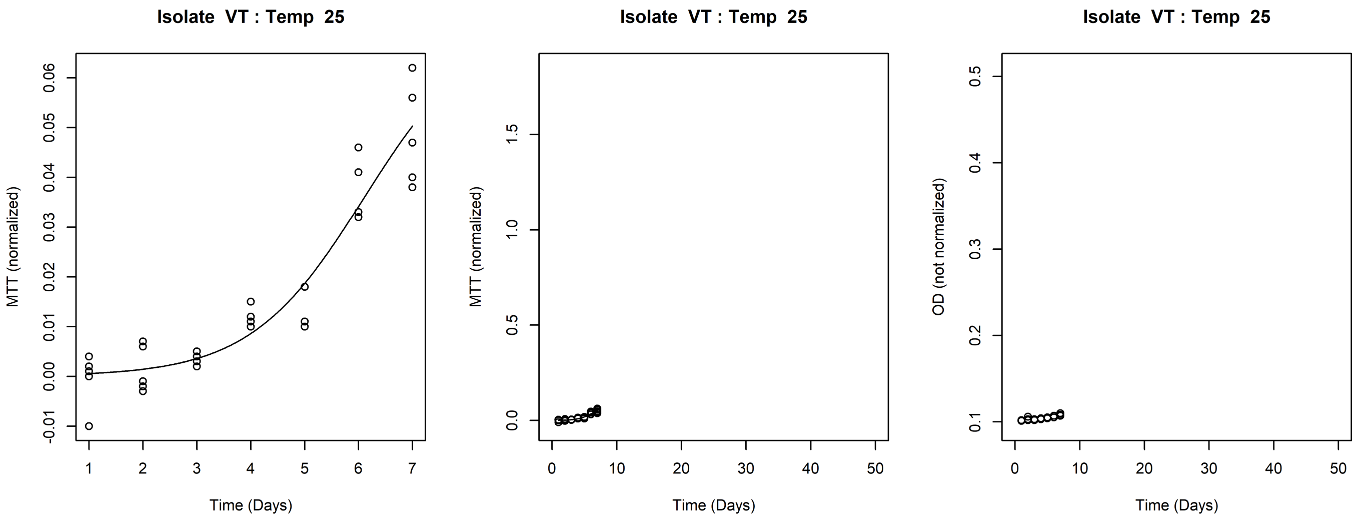


```
##           x0      r       K       b0     b1 convcode
## 33 0.0002178 0.9506 0.07155 0.003641 0.1023        0
```

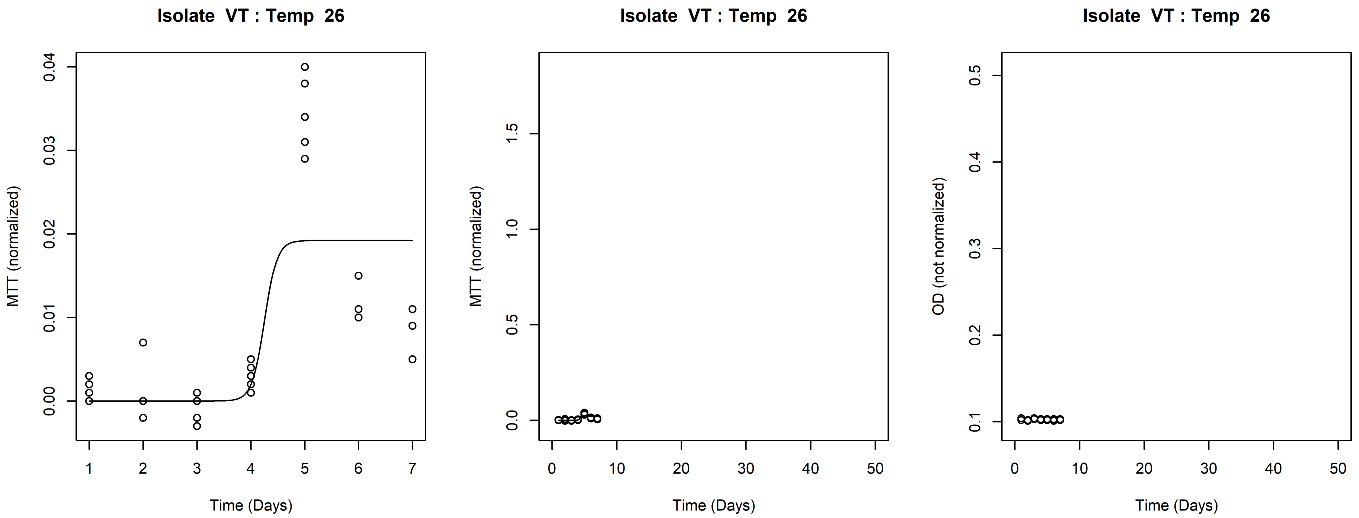


```
##           x0     r       K       b0     b1 convcode
## 34 2.719e-18 8.583 0.01922 0.002236 0.4546        0
```

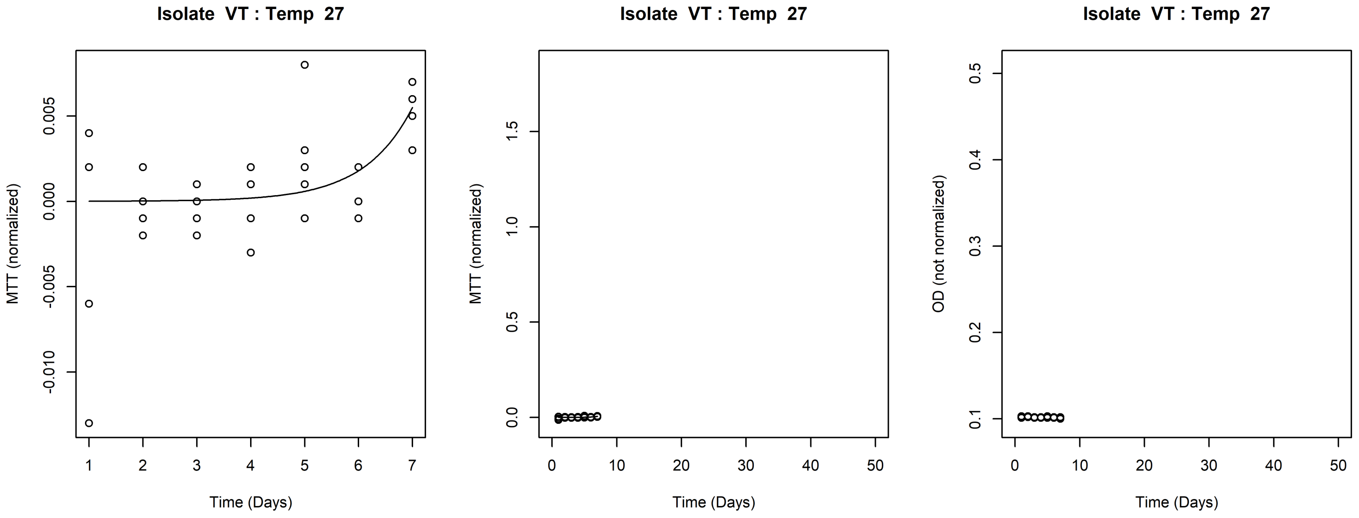


```
##           x0     r     K       b0       b1 convcode
## 35 2.217e-06 1.117 42.44 0.003122 3.36e-10        0
```

95% Confidence Intervals for r and K

The plots below show the likelihood profiles for each temperature-isolate combination, first for r and then for K. The dashed red horizontal line is the cutoff that determines the 95% confidence interval (see above) and the dotted gray horizontal line shows the cutoff that determines the 99% confidence interval. The lower and upper bounds that define the 95% confidence interval are indicated with blue dashed vertical lines. These likelihood profiles also show cases where a parameter may not be uniquely estimable, e.g., some cases where only exponential growth phase data were collected do not contain sufficient information in the data to estimate K, they only contain enough information to provide a lower bound on K, as indicated by a likelihood profile that initially decreases as K is increased, then flattens instead of increasing.

Profile likelihood plots

As mentioned above, these show both the 95% confidence interval cutoff (red) as well as a 99% cutoff (gray), but the CIs are computed using the 95% threshold.

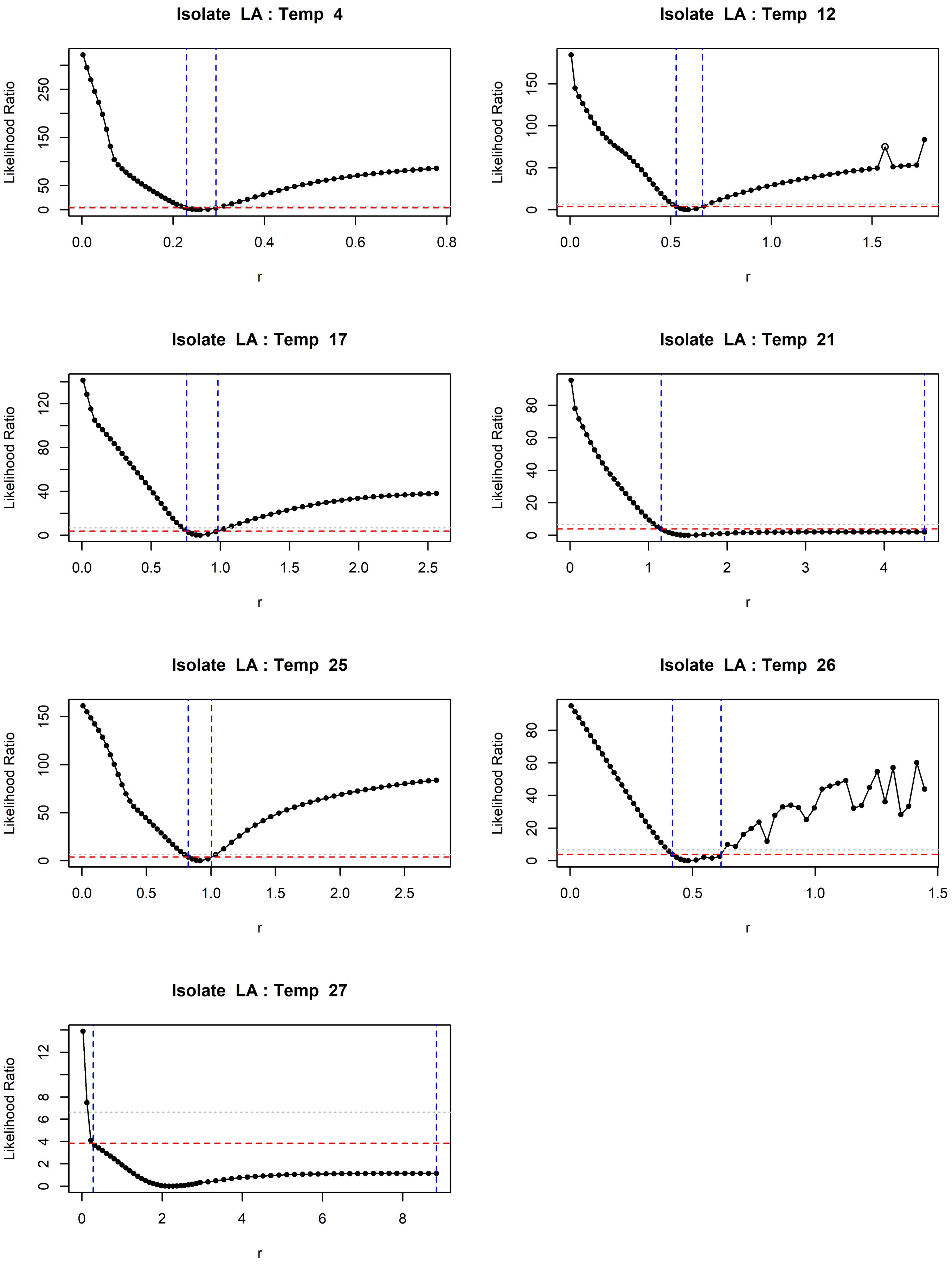
Isolate LA : Temp 4
Isolate LA : Temp 12
Isolate LA : Temp 17
Isolate LA : Temp 21
Isolate LA : Temp 25
Isolate LA : Temp 26
Isolate LA : Temp 27
Likelihood Ratio
r

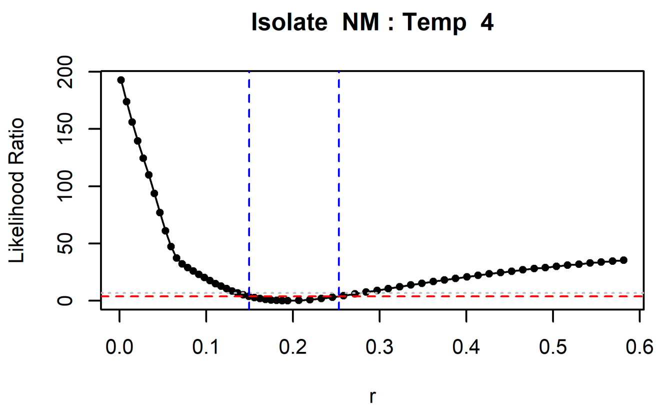
Isolate NM : Temp 4
Likelihood Ratio
0 50 100 150 200
0.0 0.1 0.2 0.3 0.4 0.5 0.6
r

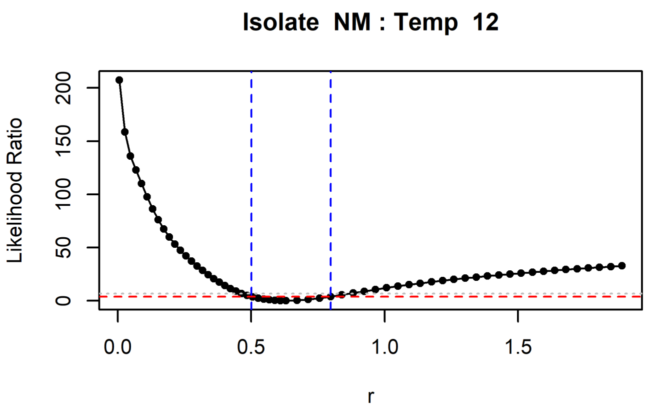
Isolate NM : Temp 12
Likelihood Ratio
0 50 100 150 200
0.0 0.5 1.0 1.5
r

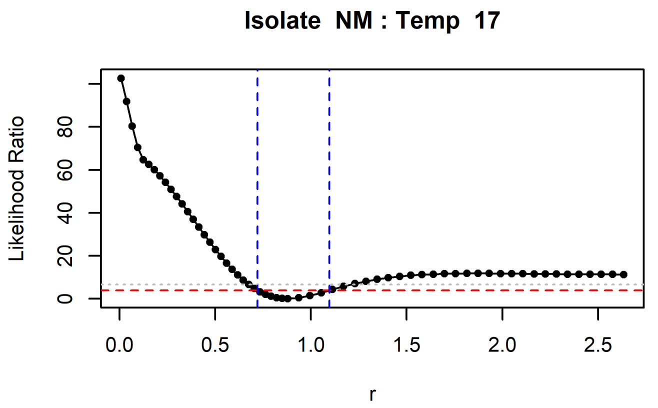
Isolate NM : Temp 17
Likelihood Ratio
0 20 40 60 80
0.0 0.5 1.0 1.5 2.0 2.5
r

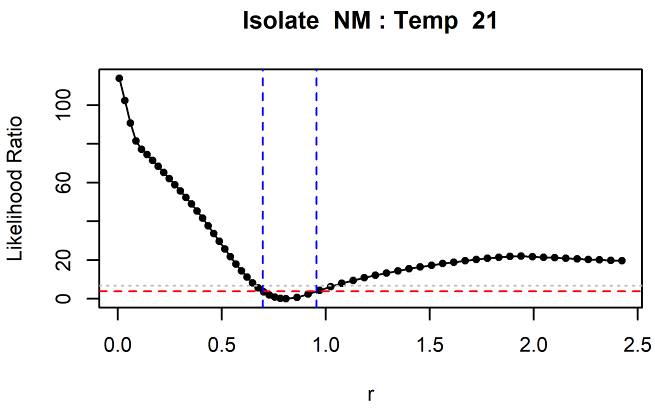
Isolate NM : Temp 21
Likelihood Ratio
0 20 60 100
0.0 0.5 1.0 1.5 2.0 2.5
r

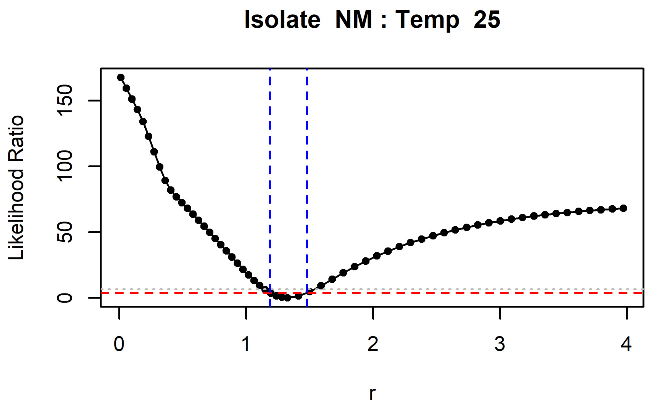
Isolate NM : Temp 25
Likelihood Ratio
0 50 100 150
0 1 2 3 4
r

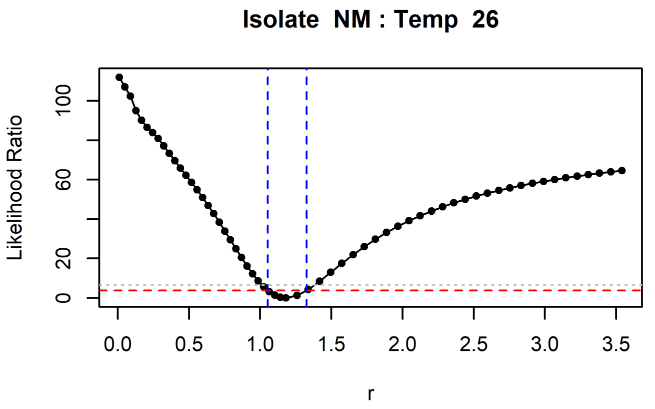
Isolate NM : Temp 26
Likelihood Ratio
0 20 60 100
0.0 0.5 1.0 1.5 2.0 2.5 3.0 3.5
r

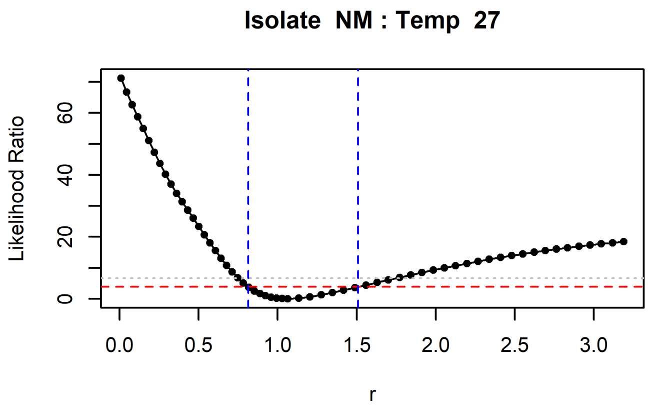
Isolate NM : Temp 27
Likelihood Ratio
0 20 40 60
0.0 0.5 1.0 1.5 2.0 2.5 3.0
r

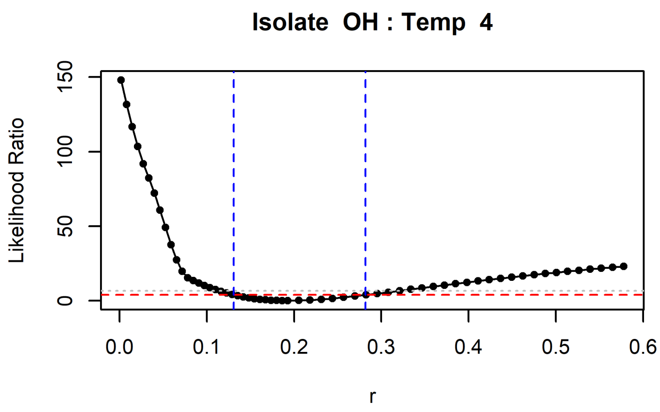
Isolate OH : Temp 4
Likelihood Ratio
0
50
100
150
0.0
0.1
0.2
0.3
0.4
0.5
0.6
r

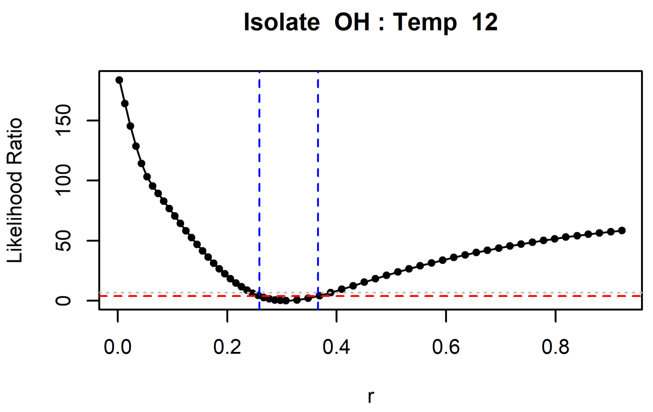
Isolate OH : Temp 12
Likelihood Ratio
0
50
100
150
0.0
0.2
0.4
0.6
0.8
r

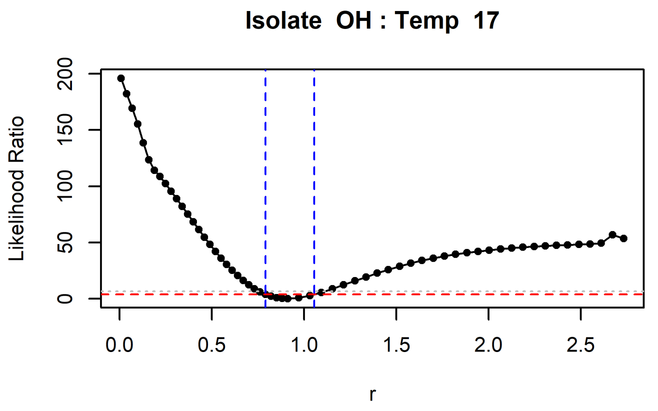
Isolate OH : Temp 17
Likelihood Ratio
0
50
100
150
200
0.0
0.5
1.0
1.5
2.0
2.5
r

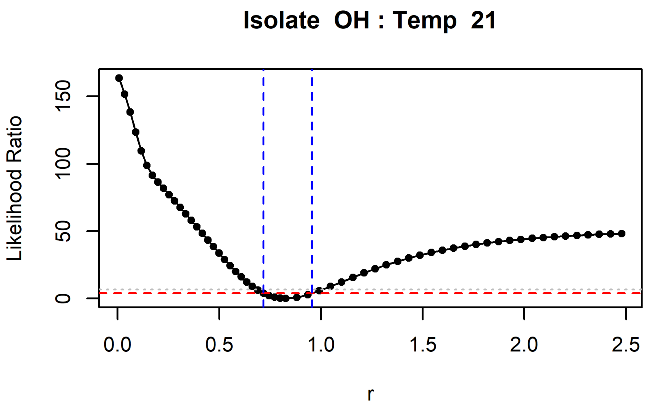
Isolate OH : Temp 21
Likelihood Ratio
0
50
100
150
0.0
0.5
1.0
1.5
2.0
2.5
r

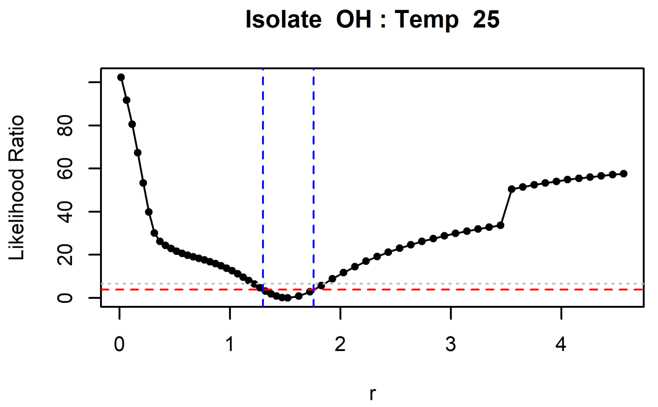
Isolate OH : Temp 25
Likelihood Ratio
0
20
40
60
80
0
1
2
3
4
r

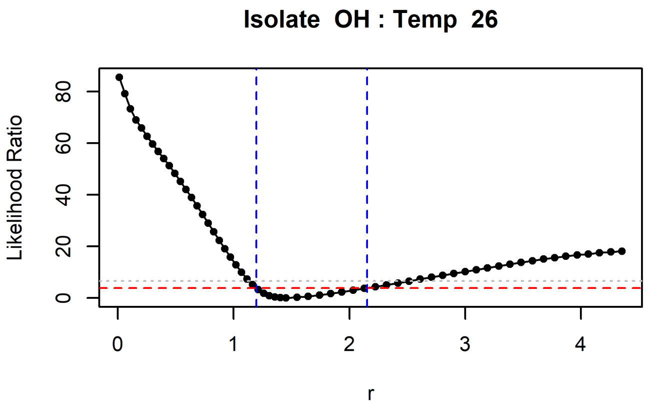
Isolate OH : Temp 26
Likelihood Ratio
0
20
40
60
80
0
1
2
3
4
r

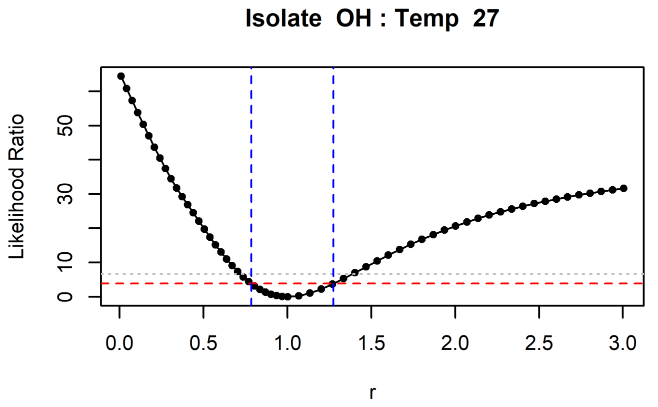
Isolate OH : Temp 27
Likelihood Ratio
0
10
30
50
0.0
0.5
1.0
1.5
2.0
2.5
3.0
r

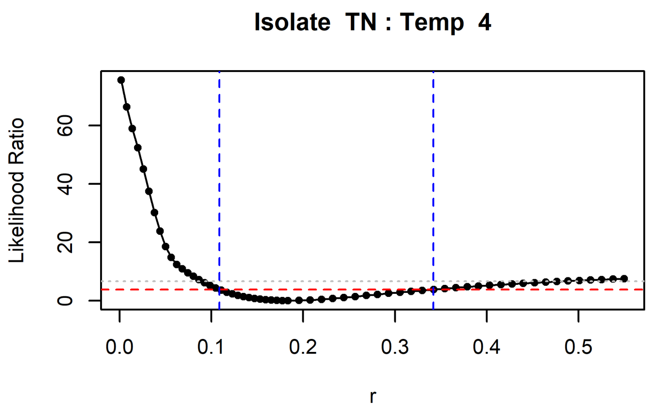
Isolate TN : Temp 4
Likelihood Ratio
0
20
40
60
0.0
0.1
0.2
0.3
0.4
0.5
r

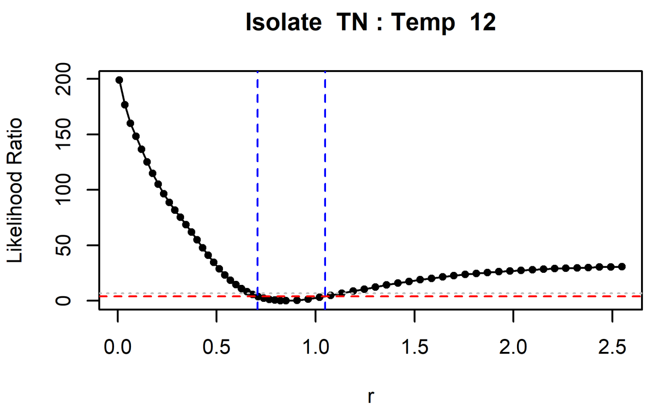
Isolate TN : Temp 12
Likelihood Ratio
0
50
100
150
200
0.0
0.5
1.0
1.5
2.0
2.5
r

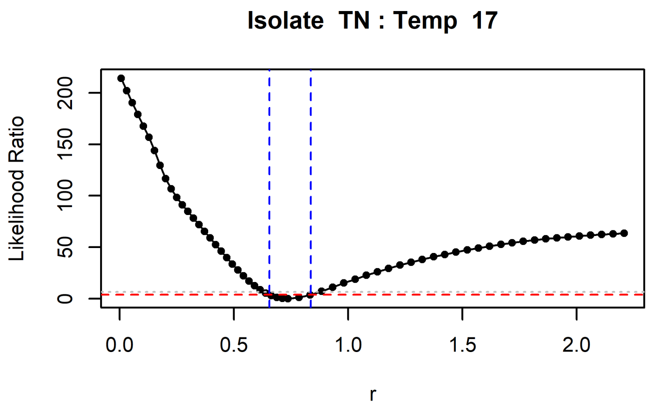
Isolate TN : Temp 17
Likelihood Ratio
0
50
100
150
200
0.0
0.5
1.0
1.5
2.0
r

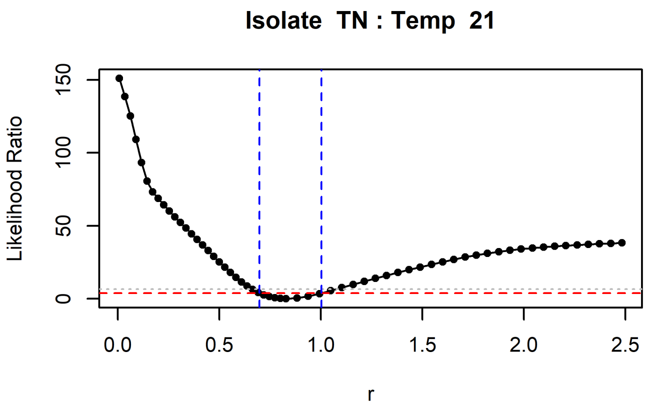
Isolate TN : Temp 21
Likelihood Ratio
0
50
100
150
0.0
0.5
1.0
1.5
2.0
2.5
r

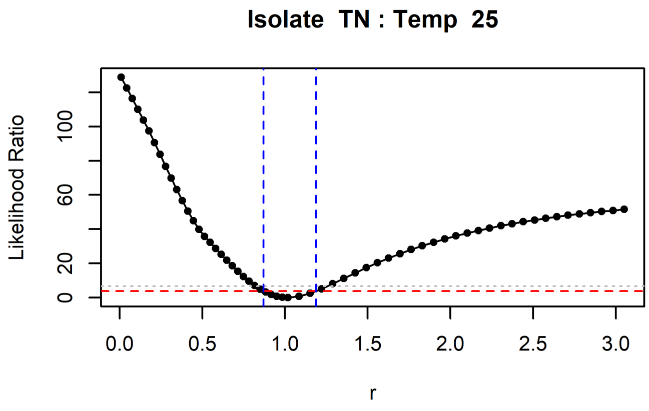
Isolate TN : Temp 25
Likelihood Ratio
0
20
60
100
0.0
0.5
1.0
1.5
2.0
2.5
3.0
r

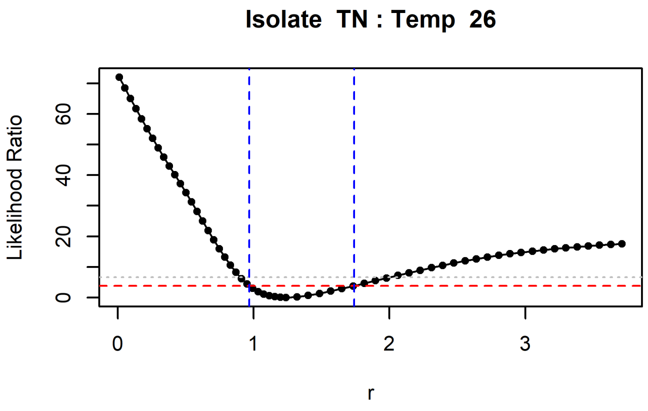
Isolate TN : Temp 26
Likelihood Ratio
0
20
40
60
0
1
2
3
r

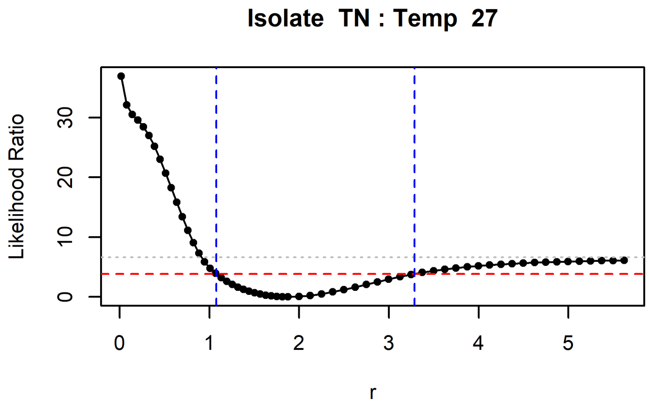
Isolate TN : Temp 27
Likelihood Ratio
0
10
20
30
0
1
2
3
4
5
r

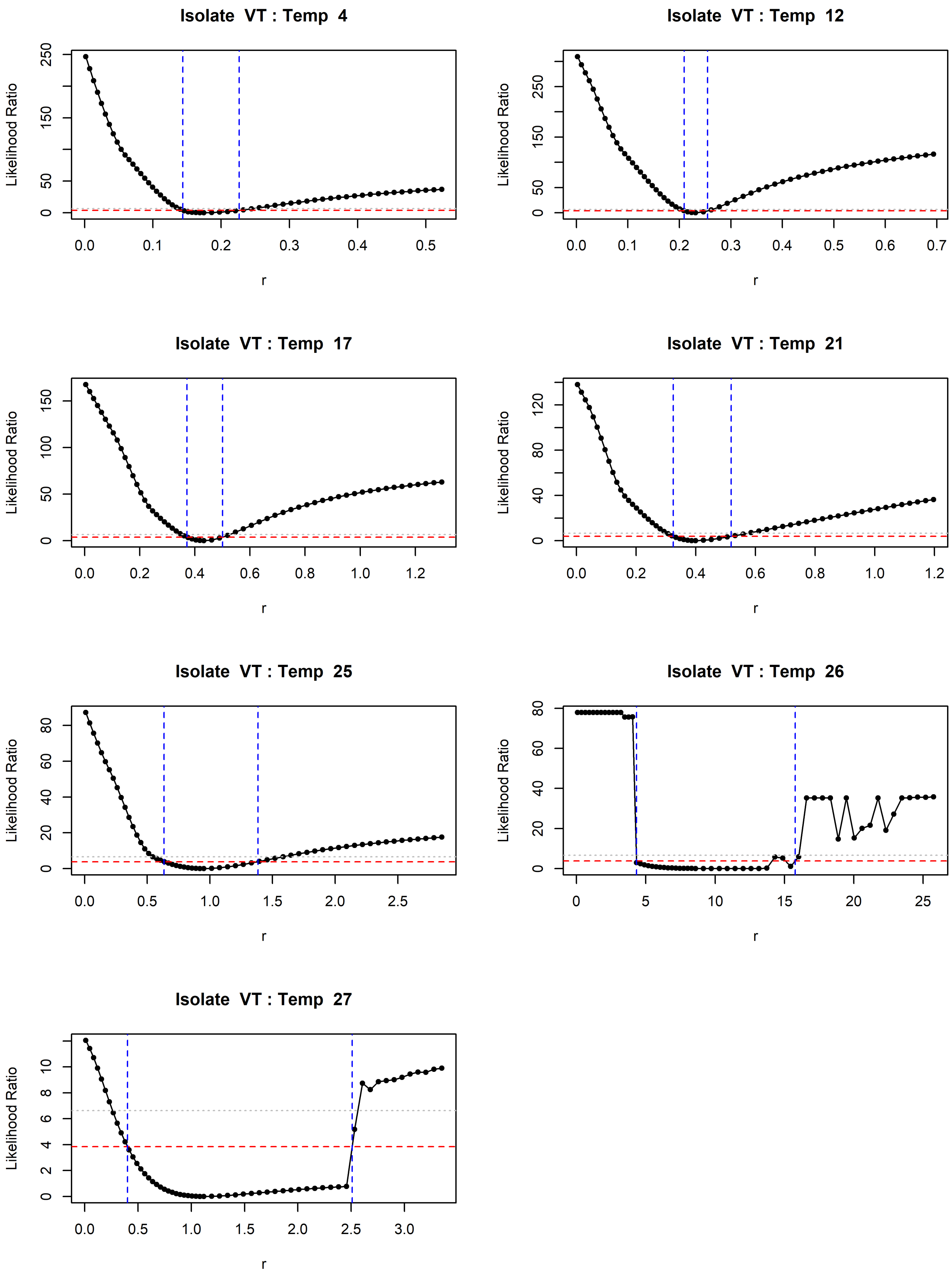
Isolate VT : Temp 4
Isolate VT : Temp 12
Isolate VT : Temp 17
Isolate VT : Temp 21
Isolate VT : Temp 25
Isolate VT : Temp 26
Isolate VT : Temp 27
Likelihood Ratio
r

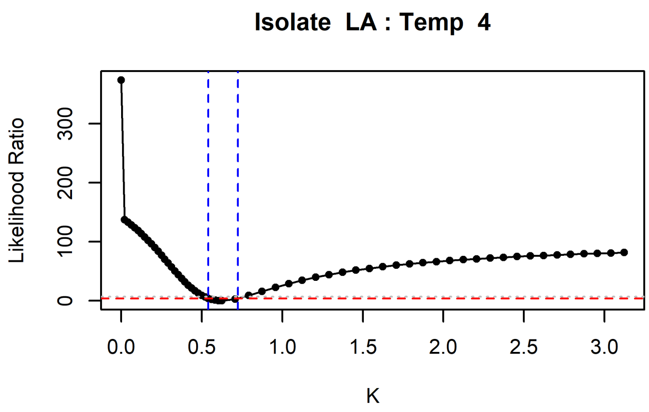
Isolate LA : Temp 4
Likelihood Ratio
0
100
200
300
0.0
0.5
1.0
1.5
2.0
2.5
3.0
K

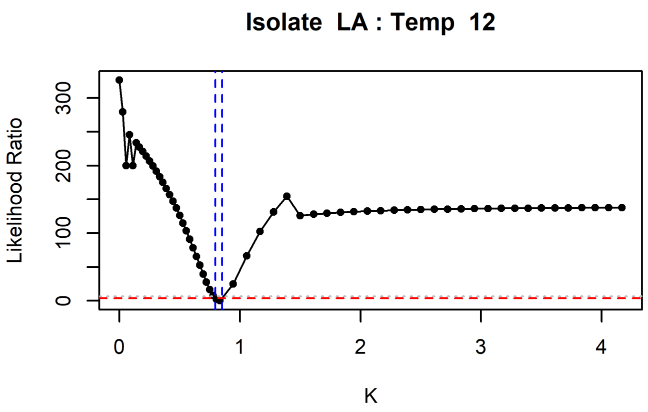
Isolate LA : Temp 12
Likelihood Ratio
0
100
200
300
0
1
2
3
4
K

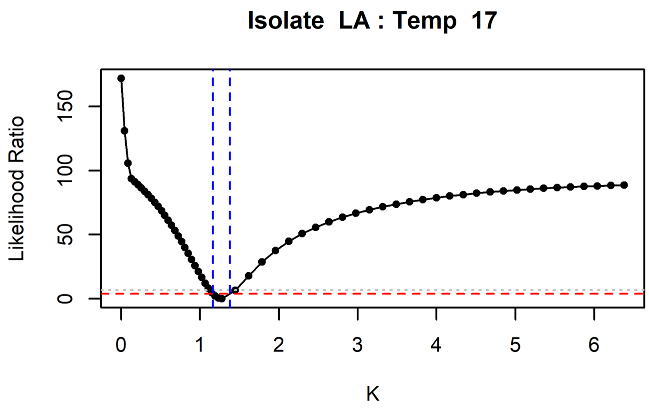
Isolate LA : Temp 17
Likelihood Ratio
0
50
100
150
0
1
2
3
4
5
6
K

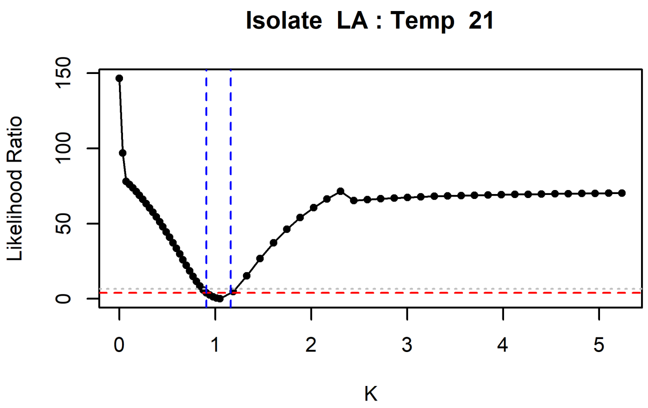
Isolate LA : Temp 21
Likelihood Ratio
0
50
100
150
0
1
2
3
4
5
K

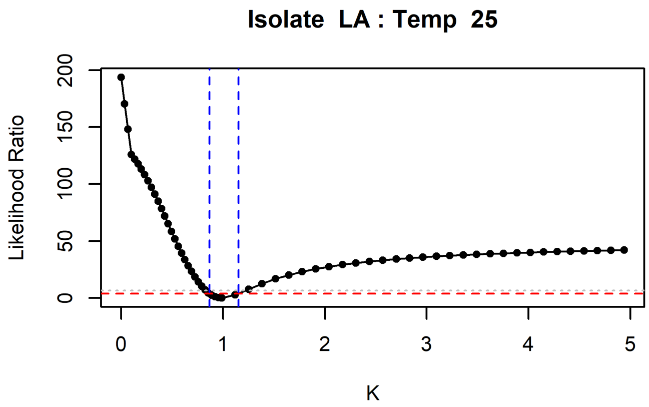
Isolate LA : Temp 25
Likelihood Ratio
0
50
100
150
200
0
1
2
3
4
5
K

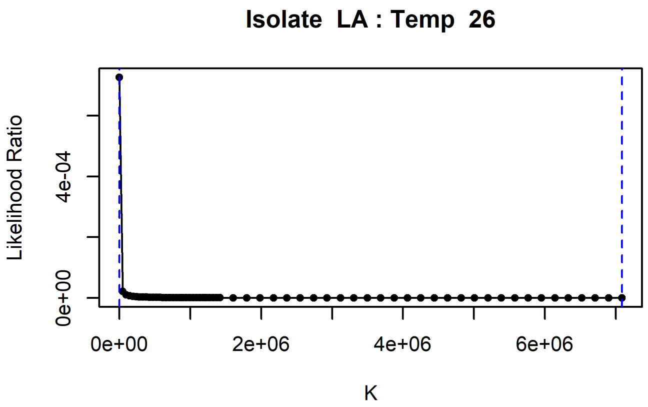
Isolate LA : Temp 26
Likelihood Ratio
0e+00
4e-04
0e+00
2e+06
4e+06
6e+06
K

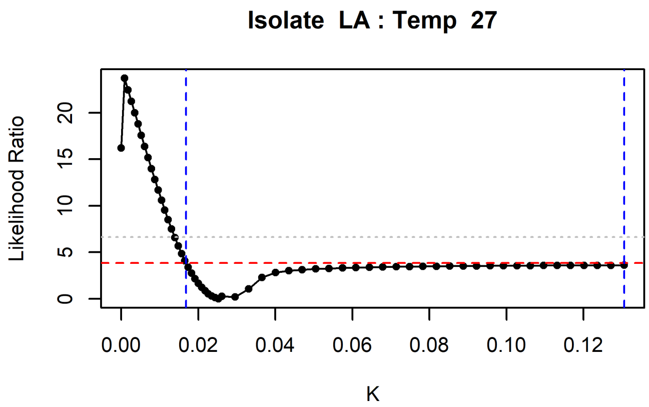
Isolate LA : Temp 27
Likelihood Ratio
0
5
10
15
20
0.00
0.02
0.04
0.06
0.08
0.10
0.12
K

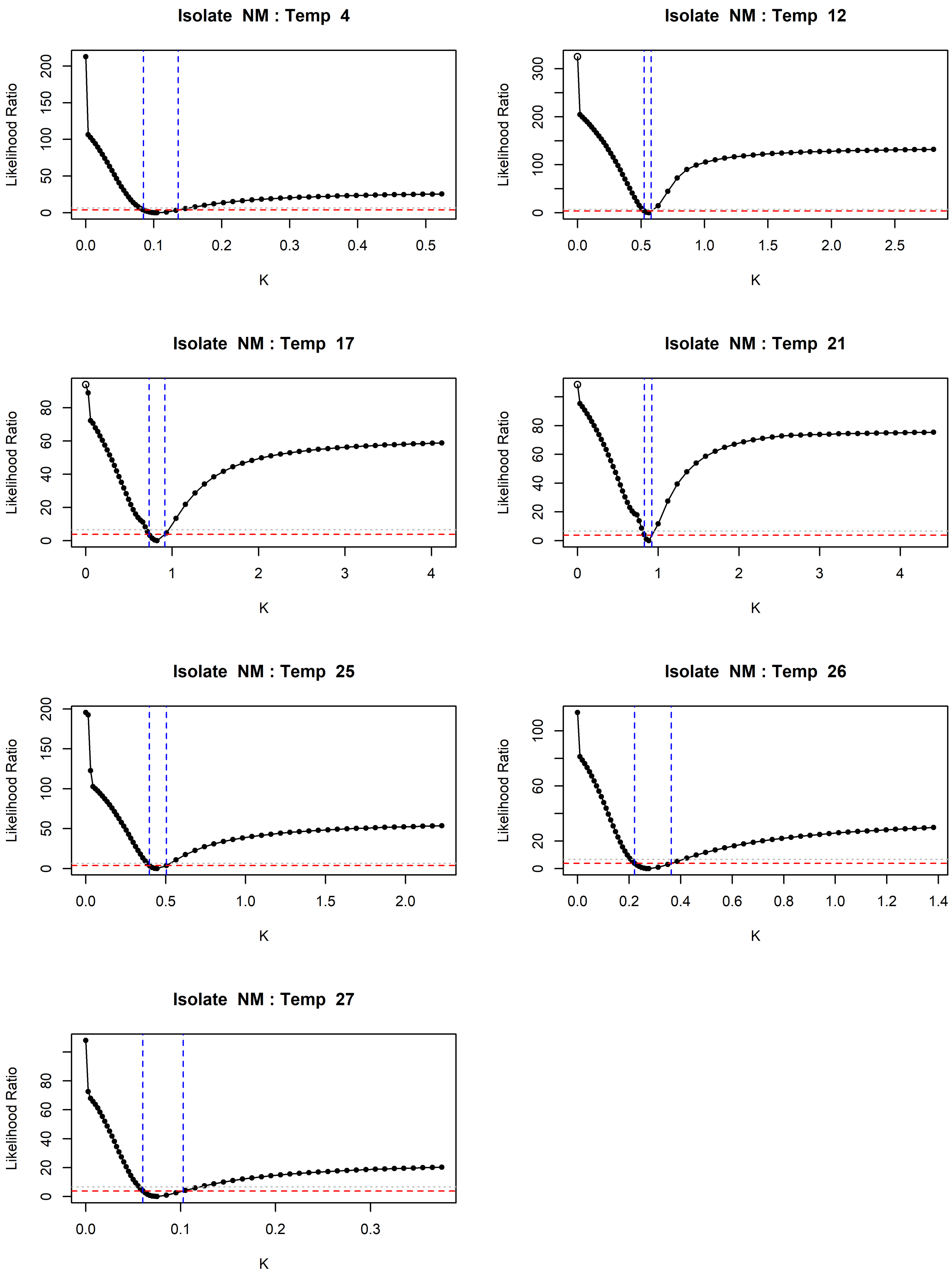
Isolate NM : Temp 4
Isolate NM : Temp 12
Isolate NM : Temp 17
Isolate NM : Temp 21
Isolate NM : Temp 25
Isolate NM : Temp 26
Isolate NM : Temp 27
Likelihood Ratio
K

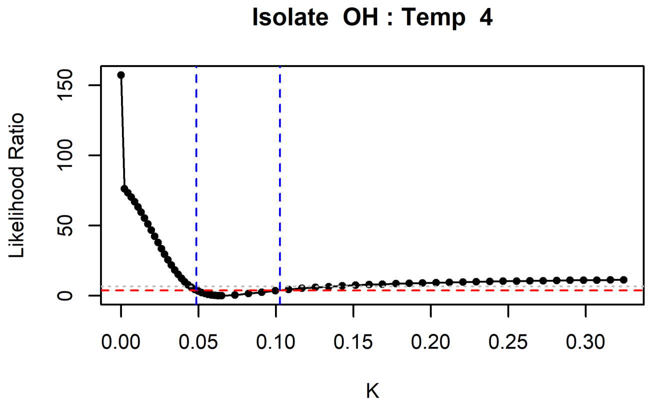
Isolate OH : Temp 4
Likelihood Ratio
K

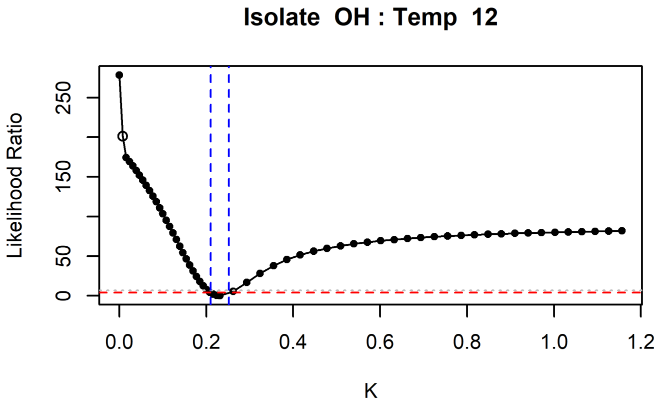
Isolate OH : Temp 12
Likelihood Ratio
K

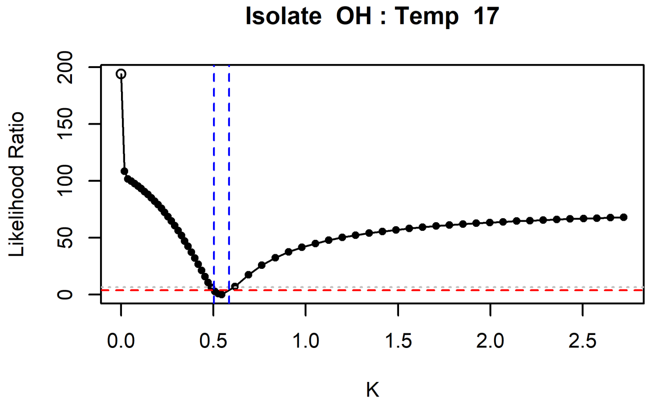
Isolate OH : Temp 17
Likelihood Ratio
K

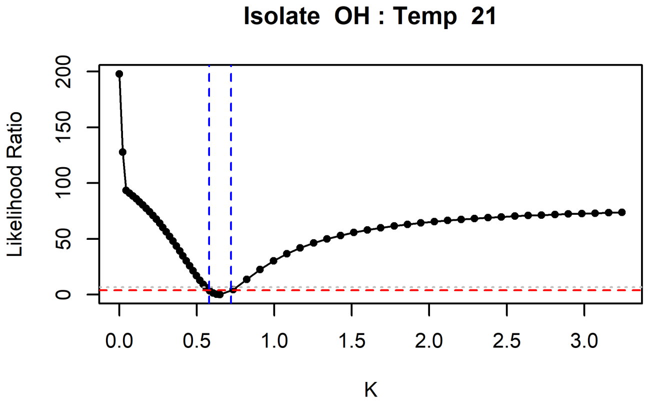
Isolate OH : Temp 21
Likelihood Ratio
K

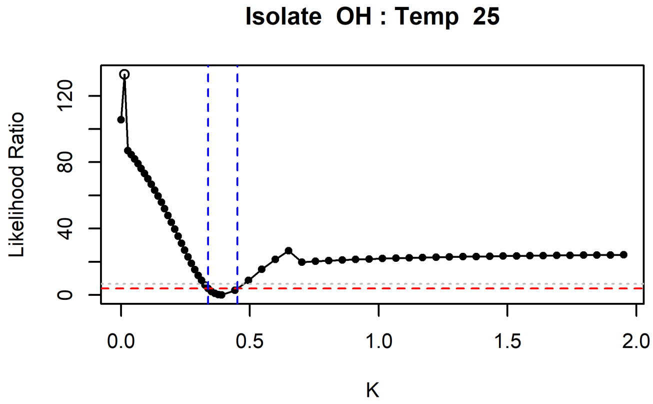
Isolate OH : Temp 25
Likelihood Ratio
K

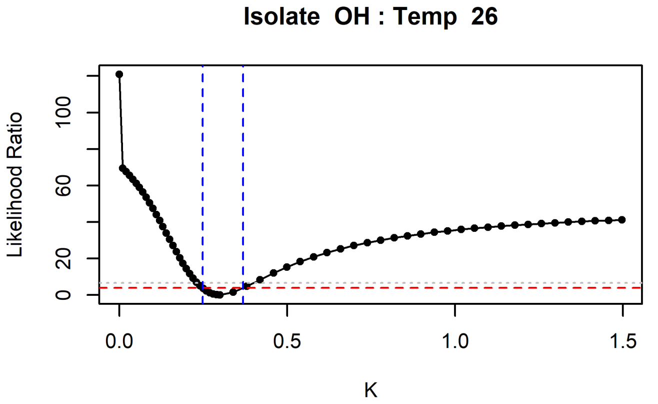
Isolate OH : Temp 26
Likelihood Ratio
K

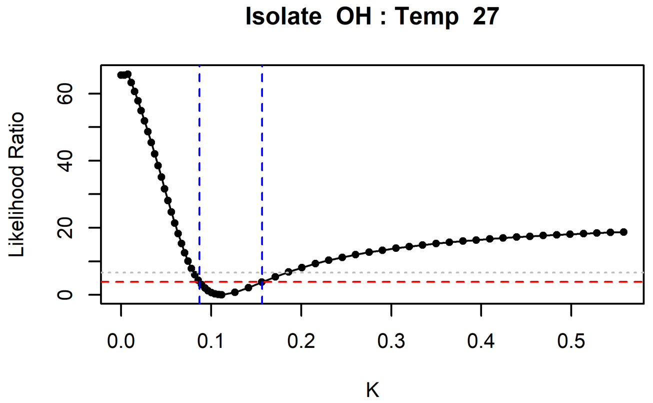
Isolate OH : Temp 27
Likelihood Ratio
K

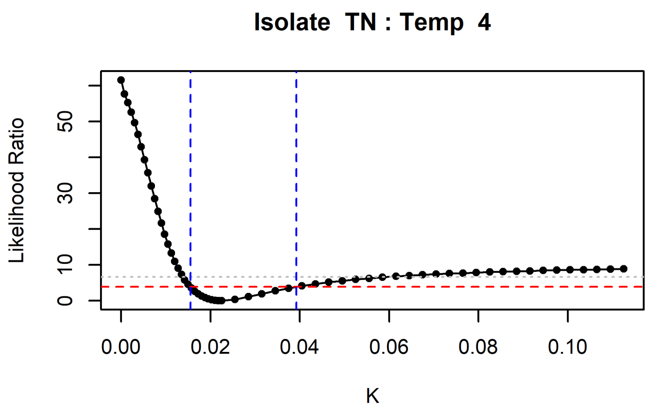
Isolate TN : Temp 4
Likelihood Ratio
0
10
30
50
0.00
0.02
0.04
0.06
0.08
0.10
K

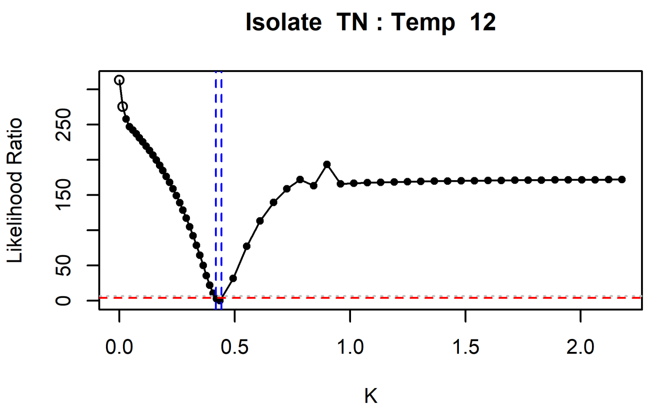
Isolate TN : Temp 12
Likelihood Ratio
0
50
150
250
0.0
0.5
1.0
1.5
2.0
K

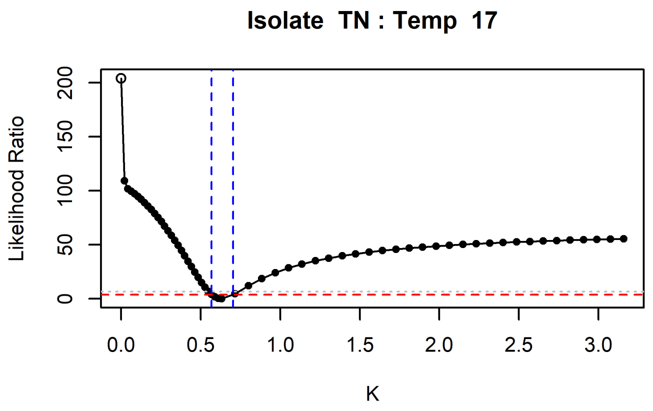
Isolate TN : Temp 17
Likelihood Ratio
0
50
100
150
200
0.0
0.5
1.0
1.5
2.0
2.5
3.0
K

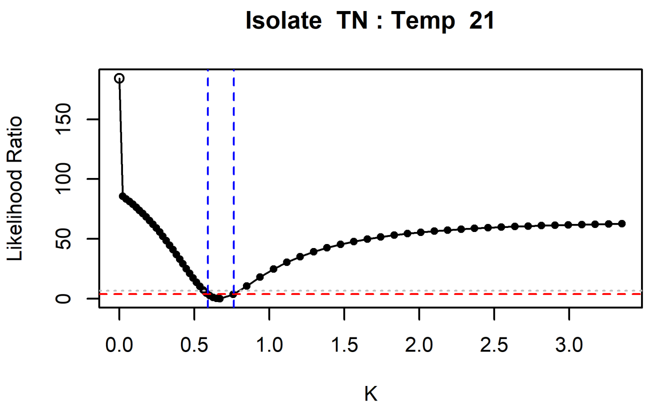
Isolate TN : Temp 21
Likelihood Ratio
0
50
100
150
0.0
0.5
1.0
1.5
2.0
2.5
3.0
K

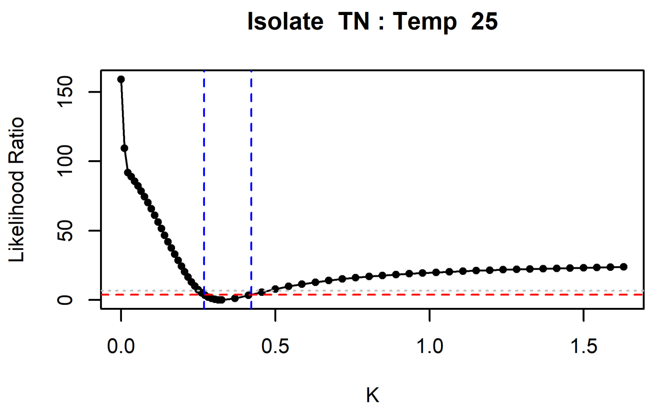
Isolate TN : Temp 25
Likelihood Ratio
0
50
100
150
0.0
0.5
1.0
1.5
K

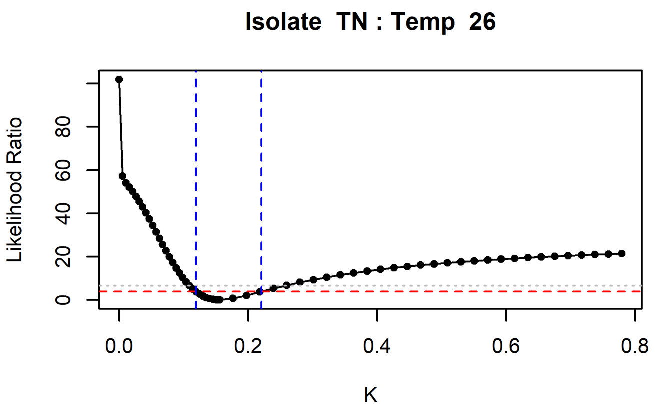
Isolate TN : Temp 26
Likelihood Ratio
0
20
40
60
80
0.0
0.2
0.4
0.6
0.8
K

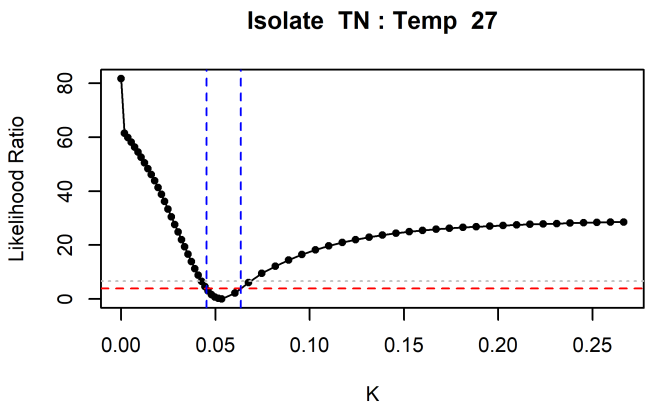
Isolate TN : Temp 27
Likelihood Ratio
0
20
40
60
80
0.00
0.05
0.10
0.15
0.20
0.25
K

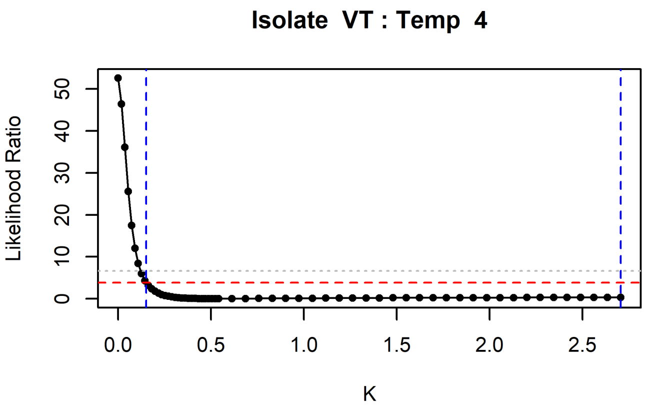
Isolate VT : Temp 4
Likelihood Ratio
0 10 20 30 40 50
0.0 0.5 1.0 1.5 2.0 2.5
K

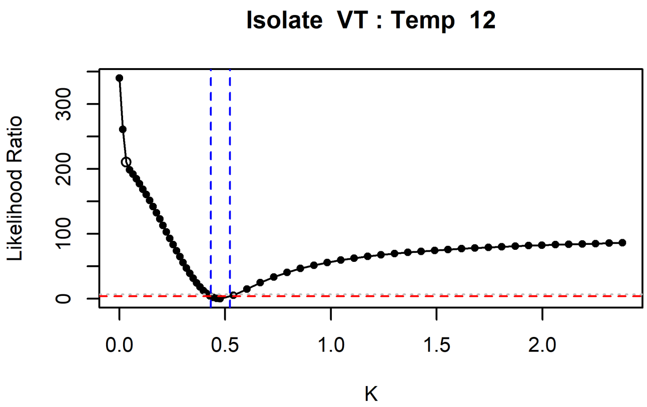
Isolate VT : Temp 12
Likelihood Ratio
0 100 200 300
0.0 0.5 1.0 1.5 2.0
K

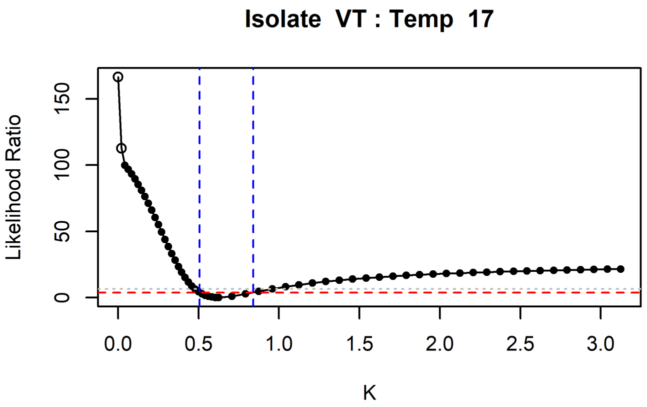
Isolate VT : Temp 17
Likelihood Ratio
0 50 100 150
0.0 0.5 1.0 1.5 2.0 2.5 3.0
K

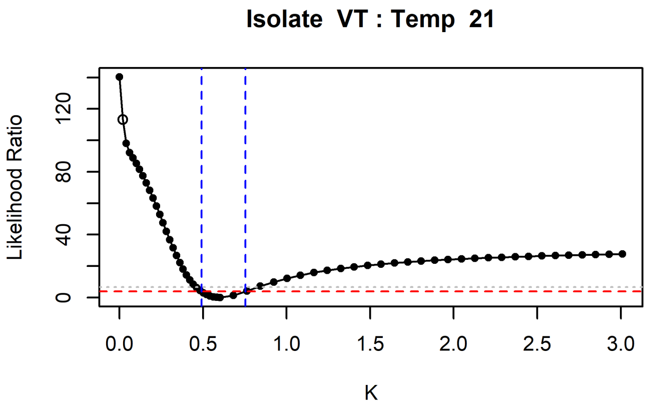
Isolate VT : Temp 21
Likelihood Ratio
0 40 80 120
0.0 0.5 1.0 1.5 2.0 2.5 3.0
K

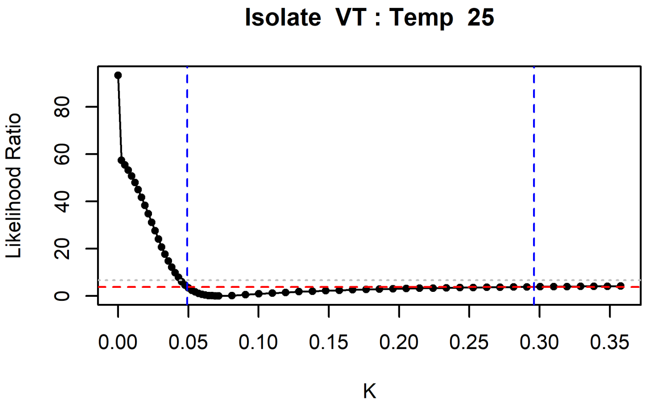
Isolate VT : Temp 25
Likelihood Ratio
0 20 40 60 80
0.00 0.05 0.10 0.15 0.20 0.25 0.30 0.35
K

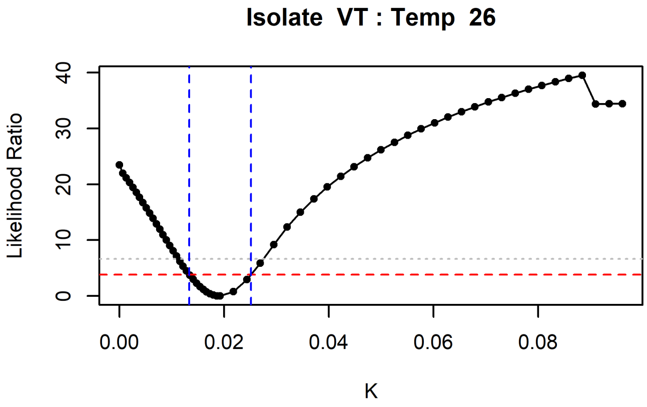
Isolate VT : Temp 26
Likelihood Ratio
0 10 20 30 40
0.00 0.02 0.04 0.06 0.08
K

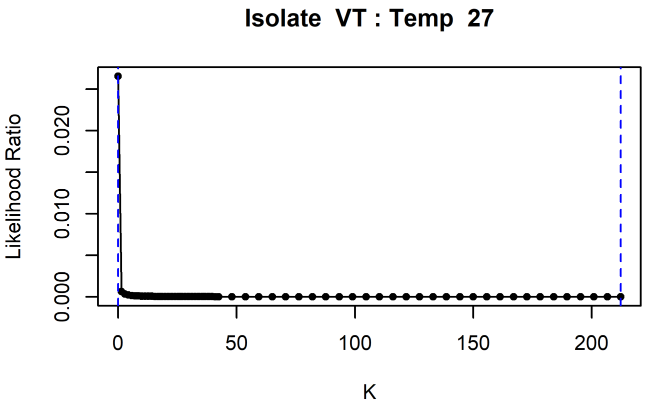
Isolate VT : Temp 27
Likelihood Ratio
0.000 0.010 0.020
0 50 100 150 200
K

```
##    isolate Temp           x0         r            K           b0           b1
## 1       LA    4 1.078206e-03 0.2590634 6.245595e-01 2.191184e-03 2.844694e-01
## 2       LA   12 7.672062e-03 0.5867231 8.337093e-01 6.476346e-03 1.616338e-01
## 3       LA   17 8.383161e-03 0.8539170 1.276013e+00 1.163975e-02 1.643746e-01
## 4       LA   21 5.453235e-03 1.5042143 1.047551e+00 7.896498e-03 2.560711e-01
## 5       LA   25 9.907636e-03 0.9155305 9.878376e-01 5.099038e-03 1.015925e-01
## 6       LA   26 1.672806e-02 0.4820032 1.417591e+06 1.610885e-02 1.997543e-01
## 7       LA   27 3.298714e-04 2.9465853 2.611439e-02 3.484856e-13 8.336986e-01
## 8       NM    4 7.017758e-04 0.1938702 1.047245e-01 3.437669e-03 2.776075e-01
## 9       NM   12 7.763511e-04 0.6296962 5.608233e-01 6.427791e-03 1.964012e-01
## 10      NM   17 1.569133e-02 0.8780131 8.240407e-01 1.190991e-02 1.799626e-01
## 11      NM   21 2.659761e-02 0.8080043 8.820931e-01 2.780935e-02 8.082133e-02
## 12      NM   25 7.644358e-04 1.3242149 4.455282e-01 2.640026e-03 1.247623e-01
## 13      NM   26 1.623522e-03 1.1802665 2.762338e-01 4.793743e-11 3.149722e-01
## 14      NM   27 1.457109e-03 1.0628639 7.503278e-02 7.448617e-04 3.302224e-01
## 15      OH    4 2.368872e-04 0.1925829 6.490205e-02 5.060213e-03 2.208802e-01
## 16      OH   12 3.522725e-03 0.3071427 2.312914e-01 4.529568e-03 2.397883e-01
## 17      OH   17 1.744314e-04 0.9106411 5.444790e-01 6.236108e-03 1.046281e-01
## 18      OH   21 1.420256e-03 0.8263378 6.487234e-01 5.343165e-03 1.851403e-01
## 19      OH   25 2.184343e-03 1.5214743 3.902704e-01 2.821321e-05 2.776008e-01
## 20      OH   26 1.868559e-03 1.4512178 2.993768e-01 1.253385e-03 3.462211e-01
## 21      OH   27 3.171476e-03 1.0015790 1.116322e-01 3.060500e-12 3.895854e-01
## 22      TN    4 1.911496e-04 0.1832258 2.250046e-02 4.518278e-03 1.836797e-01
## 23      TN   12 2.471917e-04 0.8495657 4.357259e-01 1.269300e-02 1.152454e-01
## 24      TN   17 4.291135e-04 0.7361102 6.317436e-01 4.631531e-03 1.075680e-01
## 25      TN   21 1.614565e-03 0.8276446 6.701784e-01 6.009578e-03 2.218679e-01
## 26      TN   25 2.319097e-03 1.0170940 3.259483e-01 2.191698e-03 1.932003e-01
## 27      TN   26 1.509779e-03 1.2371524 1.559253e-01 1.060328e-03 4.333080e-01
## 28      TN   27 2.421457e-04 1.8740154 5.331168e-02 3.883148e-03 2.502553e-01
## 29      VT    4 8.794540e-05 0.1744660 5.413685e-01 4.315316e-03 2.000063e-01
## 30      VT   12 4.056181e-03 0.2312362 4.757365e-01 6.644453e-03 1.262364e-01
## 31      VT   17 6.070686e-03 0.4320938 6.248107e-01 6.377395e-03 1.346735e-01
## 32      VT   21 1.689098e-02 0.3991459 6.020393e-01 1.619324e-02 9.052296e-02
## 33      VT   25 2.177900e-04 0.9505612 7.155160e-02 3.640874e-03 1.022782e-01
## 34      VT   26 2.719409e-18 8.5830803 1.922429e-02 2.235604e-03 4.546393e-01
## 35      VT   27 2.217344e-06 1.1167202 4.244246e+01 3.122041e-03 3.359615e-10
##    convcode        NLL nobs
## 1         0 -161.81533   75
## 2         0  -79.21697   80
## 3         0  -41.35193   38
## 4         0  -24.35651   38
## 5         0  -73.27635   35
## 6         0  -59.99346   35
## 7         0  -80.68266   30
## 8         0 -236.96651   75
## 9         0 -110.41979   80
## 10        0  -40.24652   34
## 11        0  -45.60532   35
## 12        0  -98.14405   35
## 13        0  -85.63105   35
## 14        0 -109.22662   35
## 15        0 -254.02903   75
## 16        0 -167.73740   80
## 17        0 -102.95198   40
## 18        0  -77.37027   40
## 19        0  -64.93115   35
## 20        0  -64.38445   35
## 21        0  -90.44952   35
## 22        0 -274.69497   74
## 23        0 -135.04664   80
## 24        0 -109.88249   40
## 25        0  -69.06880   40
```

```
## 26        0  -92.35807   35
## 27        0  -79.81859   35
## 28        0  -94.91163   32
## 29        0 -274.12872   75
## 30        0 -187.27877   80
## 31        0  -96.00241   40
## 32        0  -78.68276   40
## 33        0 -134.44692   35
## 34        0 -137.31954   35
## 35        0 -151.15800   35
```

```
## [1] " Here are the r confidence interval limits "
```

```
##    Temp isolate    rlower     rupper found.lower found.upper
## 1     4      LA 0.2292959  0.2940164        TRUE        TRUE
## 2    12      LA 0.5263215  0.6573467        TRUE        TRUE
## 3    17      LA 0.7569545  0.9826965        TRUE        TRUE
## 4    21      LA 1.1587989  4.5126429        TRUE       FALSE
## 5    25      LA 0.8240365  1.0051841        TRUE        TRUE
## 6    26      LA 0.4176526  0.6157598        TRUE        TRUE
## 7    27      LA 0.2814622  8.8397558        TRUE       FALSE
## 8     4      NM 0.1493578  0.2529656        TRUE        TRUE
## 9    12      NM 0.5011717  0.7972850        TRUE        TRUE
## 10   17      NM 0.7212113  1.0961544        TRUE        TRUE
## 11   21      NM 0.6978039  0.9555013        TRUE        TRUE
## 12   25      NM 1.1864515  1.4777541        TRUE        TRUE
## 13   26      NM 1.0522883  1.3259138        TRUE        TRUE
## 14   27      NM 0.8130549  1.5082925        TRUE        TRUE
## 15    4      OH 0.1309649  0.2818191        TRUE        TRUE
## 16   12      OH 0.2591165  0.3657710        TRUE        TRUE
## 17   17      OH 0.7900681  1.0556496        TRUE        TRUE
## 18   21      OH 0.7178522  0.9565726        TRUE        TRUE
## 19   25      OH 1.3001598  1.7571974        TRUE        TRUE
## 20   26      OH 1.1961378  2.1537904        TRUE        TRUE
## 21   27      OH 0.7853119  1.2736843        TRUE        TRUE
## 22    4      TN 0.1085697  0.3416453        TRUE        TRUE
## 23   12      TN 0.7071292  1.0484447        TRUE        TRUE
## 24   17      TN 0.6550232  0.8359714        TRUE        TRUE
## 25   21      TN 0.6981779  1.0033057        TRUE        TRUE
## 26   25      TN 0.8701980  1.1872033        TRUE        TRUE
## 27   26      TN 0.9682207  1.7390811        TRUE        TRUE
## 28   27      TN 1.0774341  3.2857901        TRUE        TRUE
## 29    4      VT 0.1438889  0.2265475        TRUE        TRUE
## 30   12      VT 0.2093787  0.2548583        TRUE        TRUE
## 31   17      VT 0.3714102  0.5005735        TRUE        TRUE
## 32   21      VT 0.3246974  0.5187218        TRUE        TRUE
## 33   25      VT 0.6343968  1.3832665        TRUE        TRUE
## 34   26      VT 4.3311781 15.7787992        TRUE        TRUE
## 35   27      VT 0.4023632  2.5086527        TRUE        TRUE
```

```
## [1] " Here are the K confidence interval limits "
```

```
##    Temp isolate       Klower       Kupper found.lower found.upper
## 1     4      LA 5.412239e-01 7.237419e-01        TRUE        TRUE
## 2    12      LA 7.969333e-01 8.509523e-01        TRUE        TRUE
## 3    17      LA 1.163373e+00 1.377908e+00        TRUE        TRUE
## 4    21      LA 9.081032e-01 1.161460e+00        TRUE        TRUE
## 5    25      LA 8.663331e-01 1.150793e+00        TRUE        TRUE
## 6    26      LA 1.417591e+03 7.087956e+06       FALSE       FALSE
## 7    27      LA 1.686124e-02 1.305719e-01        TRUE       FALSE
## 8     4      NM 8.473013e-02 1.360709e-01        TRUE        TRUE
## 9    12      NM 5.247461e-01 5.802449e-01        TRUE        TRUE
## 10   17      NM 7.337385e-01 9.165345e-01        TRUE        TRUE
## 11   21      NM 8.272623e-01 9.207185e-01        TRUE        TRUE
## 12   25      NM 3.985363e-01 5.042385e-01        TRUE        TRUE
## 13   26      NM 2.211046e-01 3.638817e-01        TRUE        TRUE
## 14   27      NM 6.008805e-02 1.026350e-01        TRUE        TRUE
## 15    4      OH 4.869205e-02 1.026431e-01        TRUE        TRUE
## 16   12      OH 2.099856e-01 2.522847e-01        TRUE        TRUE
## 17   17      OH 5.028084e-01 5.846507e-01        TRUE        TRUE
## 18   21      OH 5.801681e-01 7.214135e-01        TRUE        TRUE
## 19   25      OH 3.382199e-01 4.511431e-01        TRUE        TRUE
## 20   26      OH 2.476370e-01 3.680603e-01        TRUE        TRUE
## 21   27      OH 8.703454e-02 1.566491e-01        TRUE        TRUE
## 22    4      TN 1.551995e-02 3.924279e-02        TRUE        TRUE
## 23   12      TN 4.191547e-01 4.428003e-01        TRUE        TRUE
## 24   17      TN 5.689626e-01 7.042897e-01        TRUE        TRUE
## 25   21      TN 5.900024e-01 7.637100e-01        TRUE        TRUE
## 26   25      TN 2.688694e-01 4.225117e-01        TRUE        TRUE
## 27   26      TN 1.191953e-01 2.205951e-01        TRUE        TRUE
## 28   27      TN 4.525465e-02 6.348513e-02        TRUE        TRUE
## 29    4      VT 1.519550e-01 2.706842e+00        TRUE       FALSE
## 30   12      VT 4.314625e-01 5.231256e-01        TRUE        TRUE
## 31   17      VT 5.066912e-01 8.402362e-01        TRUE        TRUE
## 32   21      VT 4.906162e-01 7.529875e-01        TRUE        TRUE
## 33   25      VT 4.918598e-02 2.960775e-01        TRUE        TRUE
## 34   26      VT 1.335145e-02 2.515182e-02        TRUE        TRUE
## 35   27      VT 4.244246e-02 2.122123e+02       FALSE       FALSE
```
